# Role of the Two-Component System CiaRH in the Regulation of Efflux Pump SatAB and Its Correlation with Fluoroquinolone Susceptibility

**DOI:** 10.1128/spectrum.00417-22

**Published:** 2022-05-31

**Authors:** Xia Yang, Wei Peng, Ningning Wang, Beibei Dou, Fengming Yang, Huanchun Chen, Fangyan Yuan, Weicheng Bei

**Affiliations:** a State Key Laboratory of Agricultural Microbiology, College of Veterinary Medicine, Huazhong Agricultural University, Wuhan, China; b Hubei Hongshan Laboratory, Wuhan, China; c Key Laboratory of Prevention and Control Agents for Animal Bacteriosis (Ministry of Agriculture), Institute of Animal Husbandry and Veterinary Sciences, Hubei Academy of Agricultural Sciences, Wuhan, China; d The Cooperative Innovation Center for Sustainable Pig Production, Huazhong Agricultural University, Wuhan, China; e Guangxi Yangxiang Co., Ltd., Guigang, China; Griffith University

**Keywords:** *Streptococcus suis*, two-component systems, fluoroquinolones, efflux pump, repressor

## Abstract

Streptococcus suis is an important pathogen in both pigs and humans. Although the diseases associated with S. suis can typically be treated with antibiotics, such use has resulted in a sustained increase in drug resistance. Bacteria can sense and respond to antibiotics via two-component systems (TCSs). In this study, the TCS CiaRH was identified as playing an important role in the susceptibility of S. suis to fluoroquinolones (FQs). We found that a Δ*ciaRH* mutant possessed lower susceptibility to FQs than the wild-type strain, with no observed growth defects at the tested concentrations and lower levels of intracellular drugs and dye. Proteomic data revealed that the levels of SatA and SatB expression were upregulated in the Δ*ciaRH* mutant compared with their levels in the wild-type strain. The *satA* and *satB* genes encode a narrow-spectrum FQ efflux pump. The phenomena associated with combined *ciaRH*-and-*satAB* deletion mutations almost returned the Δ*ciaRH* Δ*satAB* mutant to the phenotype of the wild-type strain compared to the phenotype of the Δ*ciaRH* mutant, suggesting that the resistance of the Δ*ciaRH* strain to FQs could be attributed to *satAB* overexpression. Moreover, SatAB expression was regulated by CiaR (a response regulator of CiaRH) and SatR (a regulator of the MarR family). The *ciaRH* genes were consistently downregulated in response to antibiotic stress. The results of electrophoretic mobility shift assays (EMSAs) and affinity assays revealed that both regulator proteins directly controlled the ABC transporter proteins SatAB. Together, the results show that cascade-mediated regulation of antibiotic export by CiaRH is crucial for the ability of S. suis to adapt to conditions of antibiotic pressure. Our study may provide a new target for future antibiotic research and development.

**IMPORTANCE** Streptococcus suis is a zoonotic pathogen with high incidence and mortality rates in both swine and humans. Following antibiotic treatment, the organism has evolved many resistance mechanisms, among which efflux pump overexpression can promote drug extrusion from the cell. This study clarified the role of CiaRH in fluoroquinolone resistance. A mutant with the *ciaRH* genes deleted showed decreased susceptibility to the antibiotics tested, an invariant growth rate, and reduced intracellular efflux pump substrates. This research also demonstrated that overexpression of the efflux pump SatAB was the main cause of Δ*ciaRH* resistance. In addition, CiaR could combine with the promoter region of *satAB* to further directly suppress target gene transcription. Simultaneously, *satAB* was also directly regulated by SatR. Our findings may provide novel insights for the development of drug targets and help to exploit corresponding inhibitors to combat bacterial multidrug resistance.

## INTRODUCTION

Streptococcus suis is a Gram-positive bacterium, with strains divided into 29 serotypes based on differences in capsule antigens (1). Generally, S. suis is considered to be spread through contact with raw, undercooked pork or internal organs (2). Surprisingly, Bonifait et al. (3) show that S. suis can be transmitted via other means, such as aerosolization. Such diverse methods of propagation reflect the potential harm of this pathogen. Thus, S. suis represents the causative microbe for various diseases in pigs (e.g., meningitis, septicemia, arthritis, endocarditis, and even sudden death), leading to serious economic loss in the global pig industry worldwide each year (4, 5). Inevitably, since 1968, when the first human case of S. suis infection was reported in Denmark, S. suis*-*associated diseases (e.g., hearing loss and meningitis) have been increasingly prevalent among humans (6, 7). In China, two large outbreaks of S. suis infections in human have also been reported (8, 9). These events have immediately aroused worldwide attention to S. suis, and antimicrobial drugs have been widely employed to prevent and treat S. suis infection in both pigs and humans.

However, antibiotic abuse has promoted antimicrobial resistance to drugs such as macrolides, lincosamides, tetracyclines, and fluoroquinolones and has become a global problem in recent years (10, 11). Thus, the prevalence of antimicrobial resistance among S. suis strains increases the risk of therapeutic failure. However, fluoroquinolones (FQs) (e.g., norfloxacin and ciprofloxacin), which function by interacting with DNA gyrase and topoisomerase IV to inhibit bacterial DNA synthesis and replication, are the recommended antibiotics for the treatment of streptococcal infections. Their frequent use has also caused increased FQ resistance (12–14). Acquired FQ resistance has been attributed to point mutations in the quinolone resistance-determining regions (QRDRs) of DNA gyrase, encoded by *gyrA* and *gyrB*, and topoisomerase IV, encoded by *parC* and *parE*, as the targets of these antibiotics (15–17). In S. suis, the target mutation hierarchy exhibits flexibility, with topoisomerase IV representing the primary target and DNA gyrase the secondary target, occasionally with contrasting outcomes (18). This finding is in line with similar reports in Streptococcus pneumoniae (18). In addition, an active efflux pump can also confer FQ resistance by increasing the drug efflux of PmrA and PatAB of S. pneumoniae (19, 20), NorA of Staphylococcus aureus (21), Bmr of Bacillus subtilis (22), and LmrA of Lactobacillus lactis (23). Similarly, as an efflux pump, SatAB has also been found to lead to FQ resistance in S. suis strain BB1013 (24). Although the above-described mechanisms of FQ resistance have been investigated extensively, few studies have focused on specific regulatory mechanisms.

Previous studies have indicated that the destabilization of transcriptional terminators can trigger high-level *satAB* expression (25). Related studies have also shown a correlation between the MarR family and the SatAB efflux pump in S. suis BB1013. MarR family proteins can regulate the expression of cognate multidrug-resistant (MDR) efflux pumps. Members of this family of proteins usually recognize the pseudopalindromic sequences of the promoter regions of target genes and act as local repressors (26). SatR is a member of the MarR family of transcriptional regulators. Its premature interruption and an Ala32Pro substitution improve the level of *satAB* expression (10- to 20-fold) and increase FQ resistance (27). However, among the 15 tested strains, homologues of the SatR regulator adjacent to the pump are only found in the five closest species (27). This phenomenon suggests that there may exist a larger and more conserved regulatory network.

Two-component regulatory systems (TCSs) usually increase bacterial adaptability to the environment. A typical TCS consists of a histidine kinase (HK) and a cognate response regulator (RR). External stimuli usually trigger the autophosphorylation of HK at a histidine (His) residue. HK subsequently transfers the same phosphate group to the aspartic acid (Asp) residue of the RR. Once phosphorylated, the RR is activated to further influence the transcription of downstream genes. CiaRH, which consists of HK CiaH and RR CiaR, is one of the best characterized TCSs and exists widely among streptococci (28). It has been associated with biofilm formation, the development of competence, virulence, β-lactam resistance, and bacteriocin production (29–33). In S. pneumoniae, CiaRH has been reported for the first time because of its involvement in cefotaxime susceptibility, and CiaH mutations like *ciaH*_C102_, *ciaH*_C202_, *ciaH*_C103_, and *ciaH*_C306_ can alter the cefotaxime susceptibility (32, 34, 35). cDNA microarray analysis shows that the expression of CiaRH is induced by vancomycin in S. pneumoniae strain TIGR4 (36). Subsequent studies reveal that CiaRH is also involved in cycloserine, bacitracin, and penicillin resistance (37–39). Furthermore, CiaRH has been reported to participate in cationic antimicrobial peptide (AMP) resistance in Streptococcus agalactiae and Streptococcus mutans (40, 41).

Our laboratory has been working on the CiaRH system for many years, studying its role in virulence in S. suis (42). It contributes to the adherence to HEp-2 and PIEC. Furthermore, CiaRH not only resists phagocytosis mediated by RAW264.7 macrophages but also enhances the survival ability in blood. More importantly, whether in CD1 mouse models or in pig models, the deletion of CiaRH can reduce mortality and morbidity. For the current work, we wondered whether the two-component regulatory system (TCS) CiaRH in S. suis was involved in the regulation of antibiotic resistance as well.

This study focused on the regulatory mechanisms of FQ resistance. We found that a deletion of the TCS CiaRH in S. suis resulted in resistance to both ciprofloxacin and norfloxacin, and we demonstrated that overexpression of the efflux pump SatAB resulted in resistance in the *ciaRH* mutant strain. In addition, our study also found that CiaRH and SatR directly regulated the expression of the pump. Furthermore, *ciaR*, *ciaH*, *satR*, *satA*, and *satB* were conserved in Streptococcus suis, which may present the conserved regulatory networks.

## RESULTS

### CiaRH influences the susceptibility of S. suis to fluoroquinolones.

The disc diffusion method was performed to obtain an antibiogram difference profile between wild-type (WT) and *ciaRH* mutant strains using commonly clinically available antibiotics, revealing that the maximum difference in sensitivity was against norfloxacin, followed by ciprofloxacin. Concretely, for both drugs, the *ciaRH* mutant strain showed a smaller diameter of the zone of inhibition than the WT strain (Fig. S1 in the supplemental materials). The MIC values of ciprofloxacin and norfloxacin for S. suis are presented in Table 1. The MIC values of ciprofloxacin and norfloxacin were 8 μg/mL and 512 μg/mL for the *ciaRH* mutant strain, compared to 1 μg/mL and 32 μg/mL for the WT strain. Significant changes were observed for the *ciaRH* mutant strain, which had an 8-fold-lower MIC for norfloxacin and a 4-fold-lower MIC for ciprofloxacin in the presence of reserpine (efflux inhibitor) compared to the untreated group and had a 4-fold-higher MIC than the WT strain. Similar results were obtained following the addition of 10 μg/mL sodium orthovanadate (ATPase inhibitor). It is also important to note that neither reserpine nor sodium orthovanadate affected the growth of the strains at the concentrations used. These results indicated that active ATP-binding cassette (ABC) efflux transporters might exist in the *ciaRH* mutant strain.

**TABLE 1:** MICs of ciprofloxacin and norfloxacin against strains with or without inhibitors

Strain description	MIC (μg/mL) of indicated drug with indicated inhibitor^*a*^					
	CIP			NOR		
	None	RES	ORT	None	RES	ORT
WT	1	0.5	0.5	32	16	16
Δ*ciaRH*	**8**	2	2	**512**	64	128
Δ*ciaRH* Δ*satAB*	0.5	0.25	0.25	16	8	8
Δ*satAB*	0.5	0.5	0.5	16	16	16
Δ*ciaR*	1	0.5	0.5	32	16	16
Δ*ciaH*	1	0.5	0.5	32	16	16
Δ*ciaRH* C*satR*	4	0.5	0.5	64	16	16
Δ*satR*	2	1	1	128	32	32
Δ*ciaRH* Δ*satR*	0.5	0.5	0.25	16	8	8
Δ*ciaRH* Δ*satR* C*satR*	8	2	2	512	64	128

a Preparations with reserpine had 20 μg/mL reserpine added, and those with sodium orthovanadate had 10 μg/mL sodium orthovanadate added. MICs are the averages of at least three determinations, which did not differ by more than 2-fold dilution. Boldface indicates a significant increase in MIC. CIP, ciprofloxacin; RES, reserpine; ORT, sodium orthovanadate; NOR, norfloxacin.

### SatA and SatB are overexpressed in the *ciaRH* mutant strain.

**FIG 1:**
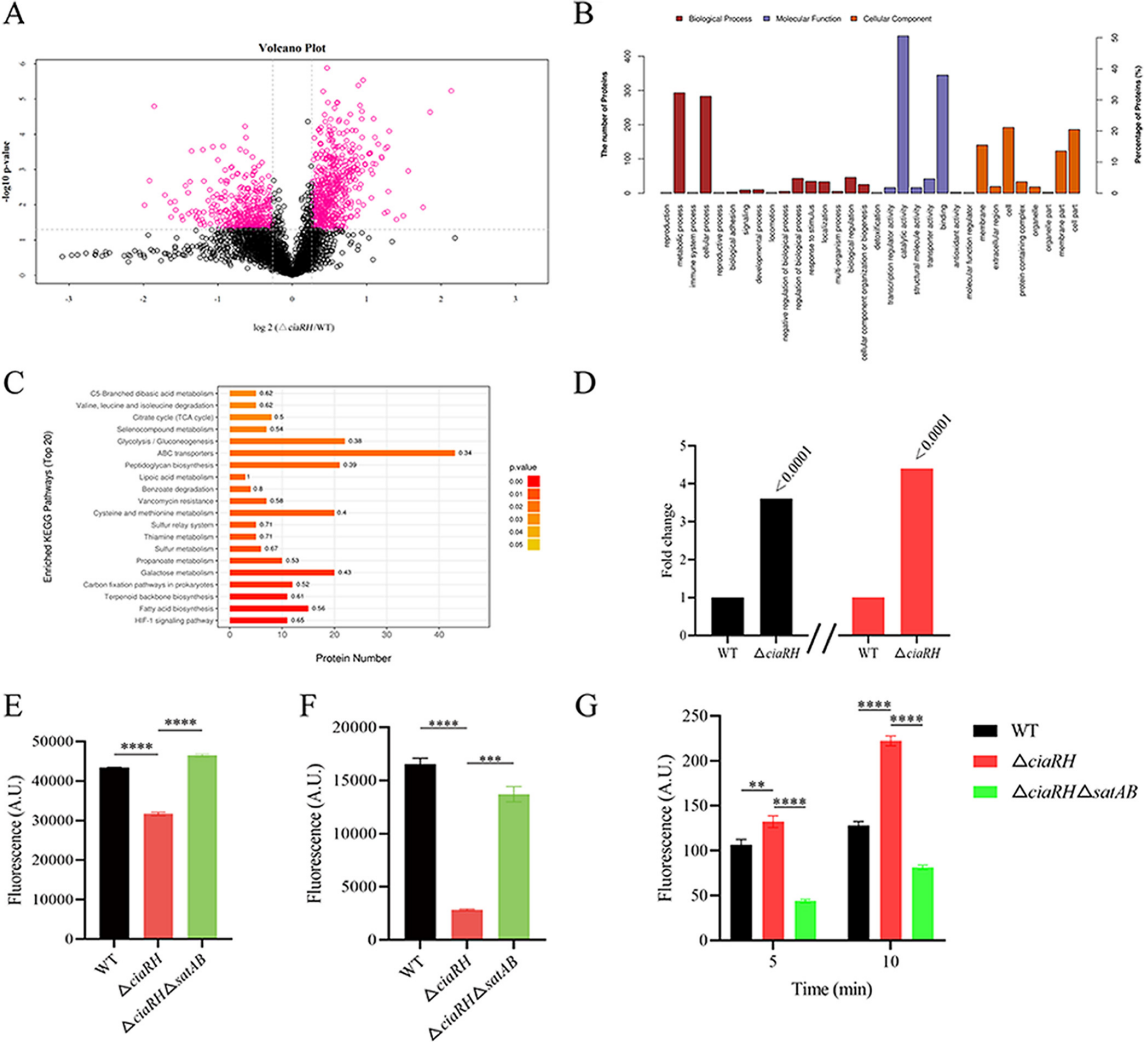
Enhanced resistance of the *ciaRH* mutant compared to the wild-type (WT) strain requires overexpression of *satAB*. (A) Volcano plot showing gene expression in Δ*ciaRH* strain versus WT strain, determined based on proteomic analysis. The *x* axis represents the log_2_(fold change value), while the *y* axis displays the −log_10_(*P* value). Red dots represent genes with 1.2-fold-higher expression in the Δ*ciaRH* strain than in the WT strain with a *P* value of <0.05, and black dots represent genes with no differential expression between the Δ*ciaRH* strain and the WT strain. (B) Gene Ontology (GO) functional enrichment analysis of DEPs. The *x* axis represents the enriched GO functional classification, whereas the *y* axis represents the number of differential proteins under each functional category. (C) KEGG pathway enrichments. The ordinate of the chart is the functional classification, and the abscissa is the protein number in a certain functional class. (D) Analysis of the expression of SatA and SatB in S. suis strains under normal growth conditions from LC-MS/MS. The data are expressed as the relative quantity of protein normalized to the proteome of the WT strain. Black columns represent values for SatA, and red columns represent values for SatB. (E) Assay of ethidium accumulation by whole cells of S. suis. Cells were incubated with ethidium bromide at a final concentration of 10 μg/mL. (F) Assay of norfloxacin accumulation. Incorporation of norfloxacin into WT, Δ*ciaRH*, and Δ*ciaRH* Δ*satAB* strains. (G) Efflux of norfloxacin. Strains were incubated at 37°C for 5 min to be preloaded with norfloxacin (512 μg/mL). A.U., arbitrary units. Error bars show standard deviations. ****, *P < *0.0001; ***, *P < *0.001; **, *P < *0.01.

Since the MIC data suggested that an efflux pump might be involved in ciprofloxacin and norfloxacin resistance, protein changes between WT and *ciaRH* mutant strains were measured at the proteome level by liquid chromatography-tandem mass spectrometry (LC-MS/MS). According to the data obtained, CiaRH could be considered a global regulator and had influenced more than 900 proteins with fold changes greater than 1.2-fold and less than 0.83-fold (*P* < 0.05) (Fig. 1A and Table S1). These differentially expressed proteins (DEPs) were found in almost every functional group and divided into three categories based on Gene Ontology (GO) analysis: biological process, molecular function, and cellular component, for which the most representative terms were metabolic process, catalytic activity, and cell, respectively (Fig. 1B). In addition, significantly greater changes in the protein abundances of ABC transporters were also identified in KEGG pathway analysis (Fig. 1C). Notably, the ABC transporter complex SatAB, which was associated with FQ resistance (24), presented with higher abundances in the *ciaRH* mutant (3.6-fold for SatA and 4.4-fold for SatB) than in the WT strain (Fig. 1D).

A previous study suggested that SatAB overexpression might contribute to increased norfloxacin and ciprofloxacin efflux in S. suis, which was demonstrated with a knock-in strategy (24). We further confirmed that this finding was also true for the *ciaRH* mutant strain. To this end, we selected two representative substrates, ethidium bromide and norfloxacin, to test whether cells overexpressing the *satAB* genes exhibited the efflux activities of these two substrates. Efflux activity was determined based on the distinction in intracellular accumulations of the substrates between the WT and *ciaRH* mutant strains. For ethidium bromide, there was a significantly lower fluorescence value in the *ciaRH* mutant strain overexpressing *satAB*, representing significantly reduced accumulation of ethidium bromide in comparison with that of the WT strain (Fig. 1E). The intracellular accumulation of norfloxacin in S. suis and its derivatives in the *ciaRH* mutant strain was monitored by fluorescence. The norfloxacin content was found to be substantially higher in the WT strain than in the *ciaRH* mutant strain. In particular, the *ciaRH* mutant strain accumulated about 6-fold-less norfloxacin than the WT strain after a 30-min exposure to antibiotics (Fig. 1F). Also, a higher fluorescence intensity was detected in the *ciaRH* mutant strain in the efflux assay (Fig. 1G). Cumulatively, the increased efflux capacity of the *ciaRH* mutant strain may be due to SatAB overexpression, leading to the strain’s multidrug resistance.

### SatAB overexpression in the *ciaRH* mutant confers drug resistance.

**FIG 2:**
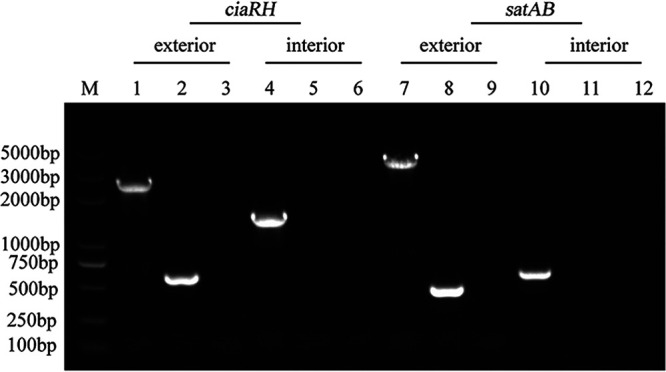
Identification of *ciaRH* and *satAB* double-gene-deletion mutant. Lane M, DL5000 DNA marker; lanes 1, 4, 7, and 10, wild type (WT) strain; lanes 2, 5, 8, and 11, Δ*ciaRH* Δ*satAB* strain; lanes 3, 6, 9, and 12, negative control; lanes 1 to 6, identification of *ciaRH* gene deletions; lanes 7 to 12, identification of *satAB* gene deletions. Primers were used as follows: lanes 1 to 3, *ciaRH*-W1 and -W2; lanes 4 to 6, *ciaRH*-IN1 and -IN2; lanes 7 to 9, *satAB*-W1 and -W2; lanes 10 to 12, *satAB*-IN1 and -IN2.

To confirm whether the resistance phenotype of the *ciaRH* mutant strain could be attributed to SatAB overexpression, we inactivated *satAB* in the mutant strain using *in vitro* mutagenesis (Fig. 2). Next, we detected the susceptibility of the double mutant to ciprofloxacin and norfloxacin and observed weakened resistance. A larger growth inhibition zone was observed for the *ciaRH satAB* double-mutant strain than for the *ciaRH* mutant strain (Fig. S2), and the ciprofloxacin and norfloxacin MIC values decreased by 16- and 32-fold, respectively, compared to those of the *ciaRH* mutant strain, reaching levels similar to those of the WT strain (Table 1). The addition of the inhibitors reserpine and sodium orthovanadate affected the MIC values of both FQ drugs moderately, by only one dilution, in this strain (Table 1). Moreover, we found that the *ciaRH satAB* double deletion mutant strain displayed markedly higher accumulations of ethidium bromide and norfloxacin and lower efflux ability for norfloxacin than the *ciaRH* mutant strain (Fig. 1E to G). In light of these data, we speculated that SatAB, as an efflux pump of both drugs, could drive FQ resistance of the *ciaRH* mutant strain.

### Bacterial growth kinetics.

**FIG 3:**
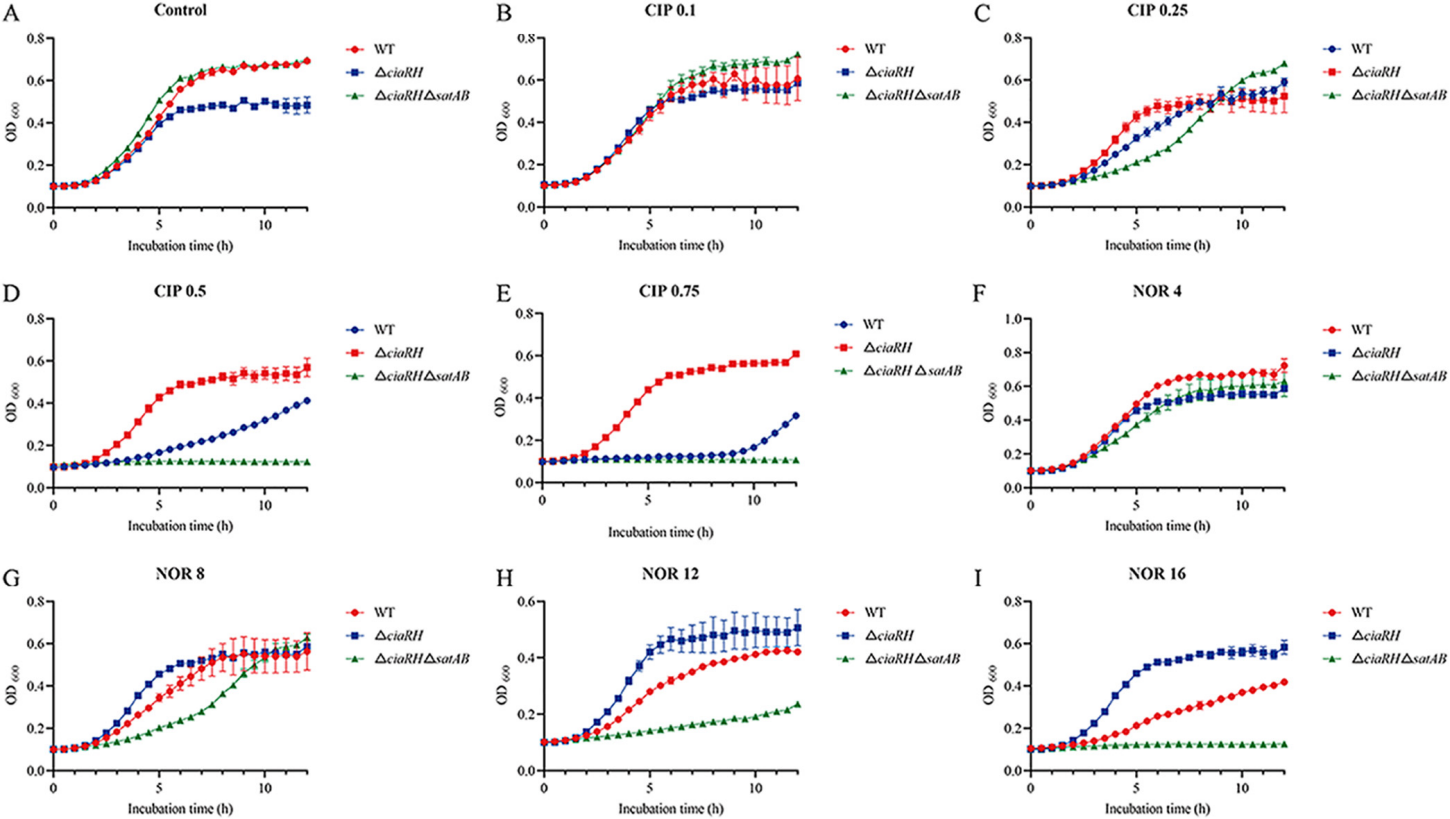
SatAB was required for S. suis resistance in liquid medium. Growth curves of the wild-type (WT), Δ*ciaRH*, and Δ*ciaRH* Δ*satAB* strains in the absence (A) and presence of antibiotic at 0.1 μg/mL (B), 0.25 μg/mL (C), 0.5 μg/mL (D), and 0.75 μg/mL (E) or 4 μg/mL (F), 8 μg/mL (G), 12 μg/mL (H), and 16 μg/mL (I). CIP, ciprofloxacin; NOR, norfloxacin; Control, no-drug treatment. The data in the graphs are the mean values and standard deviations from three wells.

To further verify the resistance potential of S. suis under FQ conditions, the growth curves of the WT, Δ*ciaRH*, and Δ*ciaRH* Δ*satAB* strains in the presence of ciprofloxacin or norfloxacin were determined by measuring the optical density of the cultures. As shown by the results in Fig. 3, the growth of the WT strain was impaired following treatment with 0.5 μg/mL ciprofloxacin and 16 μg/mL norfloxacin. However, at the same drug concentrations, the *ciaRH* mutant strain retained a normal growth rate, whereas the growth was completely limited for the *ciaRH satAB* double-mutant strain, which gradually returned to normal with decreasing drug concentrations. The differences in the growth curves were already evident during the early exponential growth phase at sub-MICs of norfloxacin and ciprofloxacin. These results were also in line with the results for MICs.

### Transcriptional start site identification of the *satRAB* operon and possible CiaR binding sites in the *satRAB* promoter regions.

**FIG 4:**
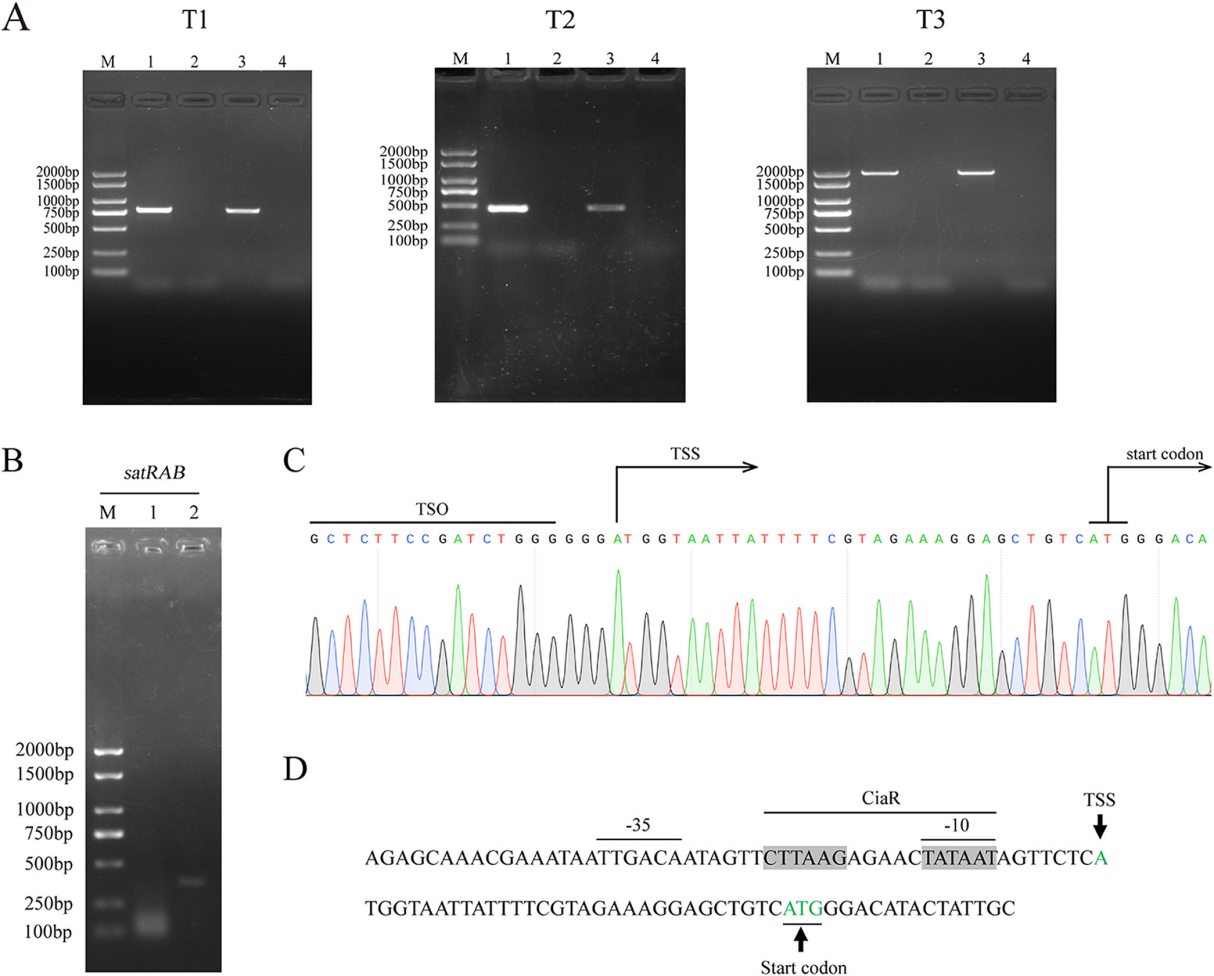
5′ RACE mapping of protein-coding genes *satRAB*. (A) RT-PCR. T1, T2, and T3 are the partial fragments of *satRA*, *satAB*, and *satRAB*, respectively. Each contains the following: lane M, DL2000 DNA marker; lane 1, cDNA; lane 2, total RNA; lane 3, genomic DNA; lane 4, no-template control. (B) Nested-PCR products of 5′ RACE starting from S. suis wild-type (WT) total RNA. Lane M, DL2000 DNA marker; lane 1, first-round PCR products obtained using the outer primer and GSP1; lane 2, second-round PCR products obtained with the inner primer and GSP2. (C) The sequencing result of the second-round PCR products. (D) Partial sequence of promoter fragment. The inferred CiaR-binding site is indicated by the gray shading. Putative −35 and −10 regions are marked with black lines. Black arrows indicate the transcription initiation site and start codon.

To identify the target sequences of the response regulator, CiaR, the transcriptional start site (TSS) of *satAB* was determined using the capping-rapid amplification of cDNA ends (capping-RACE) technique. Notably, *satR* was observed to be part of the *satRAB* operon (Fig. 4A). Thus, the TSS of the *satAB* was located upstream from *satR*. The nested-PCR products of RACE are shown in Fig. 4B. The specific second-round PCR products were purified from the gel and sequenced. According to the sequencing results, the first nucleotide base of the promoter region next to the template-switching oligonucleotide (TSO) was an adenosine residue (A), which was considered to be the TSS of the *satRAB* operon (Fig. 4C), annotated as +1.

Motif Blast was performed with the consensus CiaR box (TTTAAG-5bp-TTTAAG) in the promoter regions of *satRAB*. A similar repeat sequence with a CiaR box was observed, CTTAAGAGAACTATAAT, with three mismatches. Moreover, we found that the CiaR-binding sequence partially overlapped the putative −10 boxes in the promoter region predicted by Bprom (Fig. 4D). These results indicated that CiaR binding might occlude the access of the RNA polymerase for the initiation of transcription.

### CiaRH is a negative regulator of FQ resistance.

**FIG 5:**
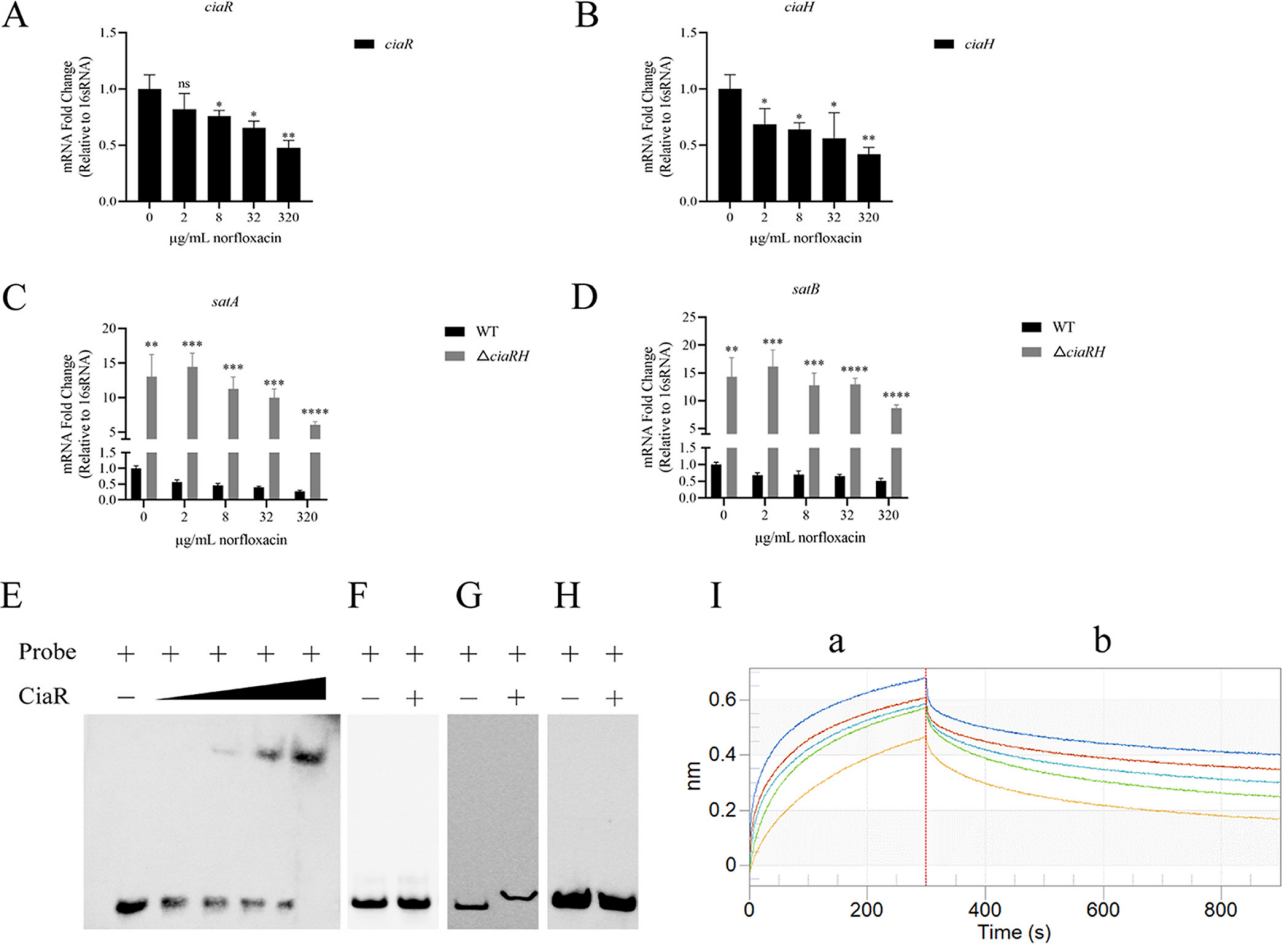
CiaRH-dependent regulation of SatAB in S. suis. (A and B) Expression of *ciaR* (A) and *ciaH* (B) in wild-type (WT) strain without or with 0, 2, 8, 32, and 320 μg/mL norfloxacin was detected by qRT-PCR. The data are expressed as the relative quantities of mRNA normalized to 16S rRNA and presented as the mean values ± standard deviations from three biological replicates. (C and D) Transcription of *satA* and *satB* in WT and Δ*ciaRH* strains without or with 0, 2, 8, 32, and 320 μg/mL norfloxacin was detected by qRT-PCR. The data are presented as described in the legend to panels A and B. (E) Electrophoretic mobility shift assay (EMSA). CiaR (0 ng, 200 ng, 400 ng, 600 ng, and 800 ng) was added to the biotin-labeled *satRAB* promoter probe. (F) EMSA of unphosphorylated CiaR protein (0 ng, 800 ng) and biotin-labeled *satRAB* promoter probe. (G and H) EMSAs. CiaR (0 ng, 800 ng) was mixed with a specific (G) and nonspecific (H) DNA. CiaR was added, where indicated, at a maximal concentration. The specific DNA was the promoter of *ciaR*, and the nonspecific DNA probe was the internal fragment of *ciaRH*. The presence or absence of probe/protein is indicated by + and −, respectively. (I) The interaction between CiaR and the promoter of the *satAB* genes was analyzed by Octet optical biosensors. The concentration of CiaR decreases from top to bottom (1, 0.5, 0.25, 0.125, and 0.0625 mg/mL). a, association; b, dissociation. The red vertical line is used to distinguish the different steps. ****, *P < *0.0001; ***, *P < *0.001; **, *P < *0.01; *, *P < *0.05; ns, not significant difference. Data were analyzed by the unpaired two-tailed Student’s *t* test.

Reasoning that the *ciaRH* mutant might have derepressed norfloxacin transport, we set out to investigate the regulation of norfloxacin transport by CiaRH. We first investigated changes in the amounts of *ciaR* and *ciaH* following the transfer of exponential-phase cells to conditions of different antibiotic concentrations. The *ciaR* and *ciaH* levels decreased as the concentration of norfloxacin increased (Fig. 5A and B), implying that the CiaRH TCS was correlated with the drug. SatAB was believed to transport ciprofloxacin and norfloxacin in the fluoroquinolone-resistant clinical S. suis isolate BB1013, based on its ability to transport both drugs when expressed in a heterologous system (24); this finding was consistent with that in the *ciaRH* mutant. Thus, we examined whether SatAB expression was induced in the presence of norfloxacin. Using quantitative real-time PCR (qRT-PCR), we measured the transcript levels of *satA* and *satB* in the WT and *ciaRH* mutant strains cultured in medium supplemented with hyper-MIC and sub-MIC levels of norfloxacin. The transcription levels of *satAB* in the *ciaRH* mutant were approximately 24-fold higher than the levels in the WT strain at 8 μg/mL norfloxacin, and similar phenomena were observed at different concentrations (Fig. 5C and D). In addition, the *satAB* genes of the *ciaRH* mutant strain were always more active than those of the WT strain.

Based on the quantitative results, it was not difficult to speculate on the remarkable effect of CiaRH on the expression of the *satA* and *satB* genes. Thus, an electrophoretic mobility shift assay (EMSA) was conducted. When the *satAB* promoter was coincubated with increasing amounts of CiaR protein, clear shifted bands were observed that were enhanced with increasing concentrations of CiaR (Fig. 5E); however, this effect was not observed using the unphosphorylated CiaR protein (Fig. 5F). The observed interactions were specific, because the CiaR protein was capable of binding to the probe consisting of the *ciaRH* promoter region but failed to bind to the unrelated probe, a *ciaRH* internal fragment, even at the maximum amount of protein (Fig. 5G and H). Similar results were obtained using an Octet RED96 system. The results of the real-time analysis showed that the affinity of CiaR and the *satAB* promoter increased along with the increasing CiaR protein concentration (Fig. 5I). The equilibrium dissociation constant (*K_D_*) value of purified CiaR binding was approximately 434 nM for the *satAB* promoter. Therefore, these findings revealed that CiaR could bind to the *satAB* promoter region and directly regulate *satAB* transcription. Also, based on the above-described results, we speculate that the decreased *ciaR* and *ciaH* transcription levels may release the inhibition of *satAB*.

### Identification of SatR as directly regulating *satAB* transcription.

**FIG 6:**
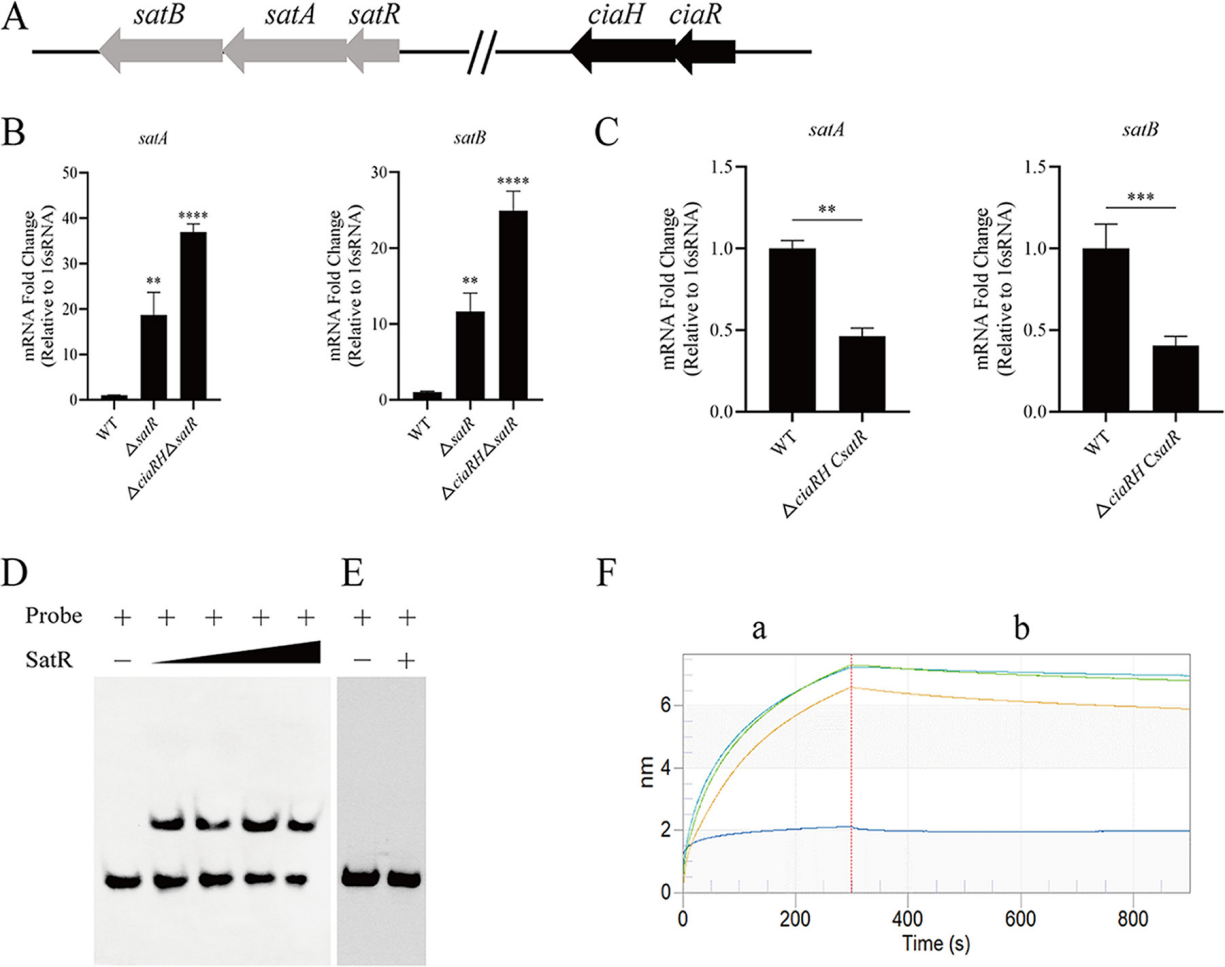
SatR directly regulated *satAB* transcription. (A) Genetic map of the *sat* genes in the wild-type (WT) strain. Open reading frames are shown by arrows. Gray arrows represent *satRAB* genes, and black arrows represent *ciaRH* genes. Arrows indicate the direction of transcription. The length and distance of arrows indicate the gene size and relative position. There are eight-base overlaps between *satR* and *satA* and between *ciaR* and *ciaH*. (B) Transcription levels of *satA* and *satB* in WT, Δ*satR*, and Δ*ciaRH* Δ*satR* strains, as determined by qRT-PCR. (C) Transcription levels of *satA* and *satB* in WT and complemented Δ*ciaRH* C*satR* strains were determined by qRT-PCR. (D) Electrophoretic mobility shift assay (EMSA). SatR (0 ng, 200 ng, 400 ng, 600 ng, and 800 ng) was incubated with biotin-labeled *satRAB* promoter probe. (E) SatR (0 ng, 800 ng) mixed with a nonspecific DNA fragment served as the negative control. The nonspecific DNA probe was an internal fragment of *satAB*. The presence and absence of probe/protein is indicated by + and −, respectively. (F) The interaction between SatR and the promoter of the *satAB* genes was analyzed by using Octet optical biosensors. The concentration of SatR decreased from top to bottom (1, 0.25, 0.125, and 0.0625 mg/mL). a, association; b, dissociation. The red vertical line is used to distinguish the different steps. Data were analyzed by unpaired two-tailed Student’s *t* test. Error bars show standard deviations. ****, *P < *0.0001; ***, *P < *0.001; **, *P < *0.01.

Consistent with the role of SatR in the clinical S. suis isolate BB1013, *satR* was cotranscribed with *satAB* and suppressed the transcription of *satAB* in the WT strain (27). As expected, *satR* was observed to be part of the *satRAB* operon, and the *satR* mutation caused significant increases in the *satAB* transcript levels (Fig. 4A and 6A and B), whereas its overexpression created notable decreases in the levels of *satAB* transcripts in the *satR*-overexpressing mutant strain (Fig. 6C). Consequently, three genes, *satR*, *satA*, and *satB*, were found to share the same promoter region. This indirectly indicated that *satR* was under the direct regulation of CiaR. To further determine whether SatR could directly regulate *satAB* transcription by binding to the promoter region of *satAB*, we performed EMSAs. SatR was found to bind to the promoter region of *satAB* (Fig. 6D), indicating that the gene was under the direct control of SatR. It was simultaneously observed that SatR was unable to bind to a nonspecific DNA fragment (Fig. 6E), even at a high concentration. Moreover, the Octet RED96 system was applied to examine the affinity and specificity of SatR for the *satAB* promoter. The affinity of the SatR protein to the target DNA increased in a dose-dependent manner (Fig. 6F). The *K_D_* value for the binding of SatR to the *satAB* promoter region was also obtained and was about 61 nM. These data suggested that SatR could regulate the transcription of *satAB* genes.

Given that the *satR* and *satAB* genes were located in the same operon and shared the same promoter, we inferred that CiaR could regulate the transcription of the *satR* and *satAB* genes. Next, we examined whether SatR had an additive effect with CiaR. We constructed a mutant with a deletion of *satR* in the Δ*ciaRH* and WT backgrounds. Unexpectedly, the *ciaRH*- and *satR*-negative double mutant was more sensitive than the *ciaRH*-negative mutant, *satR*-negative mutant, and WT strains (Table 1). This finding was contradictory to the qRT-PCR results showing that the level of *satAB* expression was obviously higher than in the WT. In addition, a complemented strain was constructed by introducing a plasmid to express *satR* in the Δ*ciaRH* Δ*satR* background. A higher MIC was obtained (Table 1) in the Δ*ciaRH* Δ*satR* C*satR* strain. These data suggest that CiaR and SatR may act through different pathways to impact FQ resistance.

### *ciaRH* and *satRAB* are widespread among S. suis isolates.

Combined with our above-described observations with S. suis, we wondered if CiaRH broadly suppressed the transcription of *satAB*. To further support this hypothesis, the nucleotide sequences of *ciaR*, *ciaH*, *satR*, *satA*, and *satB* in other clinical S. suis strains already sequenced were compared, revealing that they were widely distributed, exhibiting sequence identities of 87.67% to 100%, 91.96% to 100%, 82.26% to 100%, 85.33% to 100%, and 90.52% to 100%, respectively (Table S2). This finding indicated that the regulation of the efflux pump SatAB by the CiaRH TCS might represent a common mechanism used by bacteria to adapt to complex external environments.

## DISCUSSION

In this study, we characterized the FQ efflux phenotype of an S. suis Δ*ciaRH* mutant strain. Our findings suggested that a deletion of the *ciaRH* genes strongly decreased susceptibility to norfloxacin and ciprofloxacin. We additionally demonstrated that the resistance phenotype might be attributed to the overexpression of ATP-binding cassette (ABC) efflux transporters, which was supported by a body of evidence presented as follows. First, proteomic studies of WT and Δ*ciaRH* strains showed that SatA and SatB were upregulated in response to the absence of the *ciaRH* genes. Second, as previously reported, pump overexpression conferred an efflux phenotype, as demonstrated by the decreased accumulation of ethidium bromide and norfloxacin and increased efflux of norfloxacin in the Δ*ciaRH* strain. Third, the *ciaRH* mutant strain showed reduced susceptibility to norfloxacin and ciprofloxacin, which was partially reversible by the addition of an efflux inhibitor, reserpine, and an ATPase inhibitor, sodium orthovanadate (43). Finally, the inactivation of *satAB* in the *ciaRH* mutant background led to an increased susceptibility to norfloxacin compared to that of the *ciaRH* mutant strain (16 μg/mL versus 512 μg/mL): clear growth inhibition zones were larger in the *ciaRH satAB* double-mutant and WT strains than in the *ciaRH* mutant strain.

The efflux pump SatAB, which is equivalent to the PatAB efflux pump of Streptococcus pneumoniae, is a member of the ABC transporters, which comprise nucleotide-binding domains (NBDs) and transmembrane domains (TMDs) (44, 45). SatAB has been previously shown to be associated with multidrug resistance, and its overexpression is a reason for S. suis FQ resistance, similar to findings for S. pneumoniae and Streptococcus pseudopneumoniae (20, 24, 46). In support of this role, the deletion of *satAB* produced an effect on the MIC value of 1 dilution (2-fold more susceptible for norfloxacin and ciprofloxacin) compared to the MIC of the WT strain, indicating that there was a difference in the sensitivity between the WT and *satAB* mutant strains (Table 1). Such differences could cause a more severe growth defect phenotype at the same drug concentration compared with the growth of the WT strain (Fig. S3). Furthermore, overexpression of these two genes often occurs together in clinical isolates (47). This phenomenon may be caused by the fact that several ABC transporters in bacteria can function in the form of homo- or heterodimers (48). Thus, in S. suis, it is likely that *satA* and *satB* interact to form a heterodimer, extruding antibiotics from cells. To date, several close homologues have been implicated, such as AmphG and AmphH and NysG and NysH, with overlapping coding sequences in Streptomycetes (48). However, *patA* and *patB* are separated by a gene in S. pneumoniae (49). Differing from the above-mentioned cases, an analysis of the WT sequence showed that the encoding genes of the pump, *satA* and *satB*, approach each other with no overlap or gene spacer in S. suis (Fig. 6B), which further made linked pairs possible for *satAB*. These observations indicated that the two parts of the system might also possess different regulatory modes.

As a counterstrategy, numerous ABC transporters are often tightly linked with two-component systems (TCSs) (50), which are regarded as major sensory and regulatory systems in prokaryotes (i.e., bacterial two-component regulatory systems are capable of regulating their activities) (51). For example, carbapenem-resistant Acinetobacter nosocomialis AdeRS can activate the expression of the AdeABC efflux pump, with a similarity to the efflux pump in Acinetobacter baumannii, enhancing resistance to omadacycline, eravacycline, and tigecycline (52, 53). BasSR of Escherichia coli positively regulates the transcription of the EmrD multidrug efflux pump (54). SmeSyRy in Stenotrophomonas maltophilia can simultaneously inversely regulate the expression of the SmeYZ and SmeDEF efflux pumps (55). Listeria monocytogenes VirRS can upregulate AnrAB expression to elevate nisin resistance (56, 57). Recent research has reported that the OmpF porin and the AcrD efflux pump are under the control of CpxAR and that this process is associated with β-lactam and aminoglycoside resistance (58). Accordingly, we showed that CiaRH monitored the expression of the SatAB efflux pump, influencing the susceptibility of S. suis to norfloxacin and ciprofloxacin in a CiaR-dependent manner.

Mutations in the corresponding histidine kinase CiaH affected the level of CiaR regulon expression (32), leading to related phenotypic changes. Unexpectedly, under certain conditions, the inactivation of histidine kinase can contribute to high activity of CiaR (59), despite phosphorylated CiaR being required for target gene regulation, which indicates the existence of an alternative mechanism for its phosphorylation. Thus, we speculated that the reason genetic inactivation of *ciaH* failed to trigger obvious changes in drug sensitivity was that CiaR was active in the absence of CiaH, which might be achieved by a phosphoryl donor like AcP and a noncorresponding histidine kinase. Besides that, in this situation, another possible explanation was that CiaR-mediated regulation might be affected through posttranslational modifications like acetylation of amino acid residues and phosphorylation of serine/threonine/tyrosine residues (60–62).

**FIG 7:**
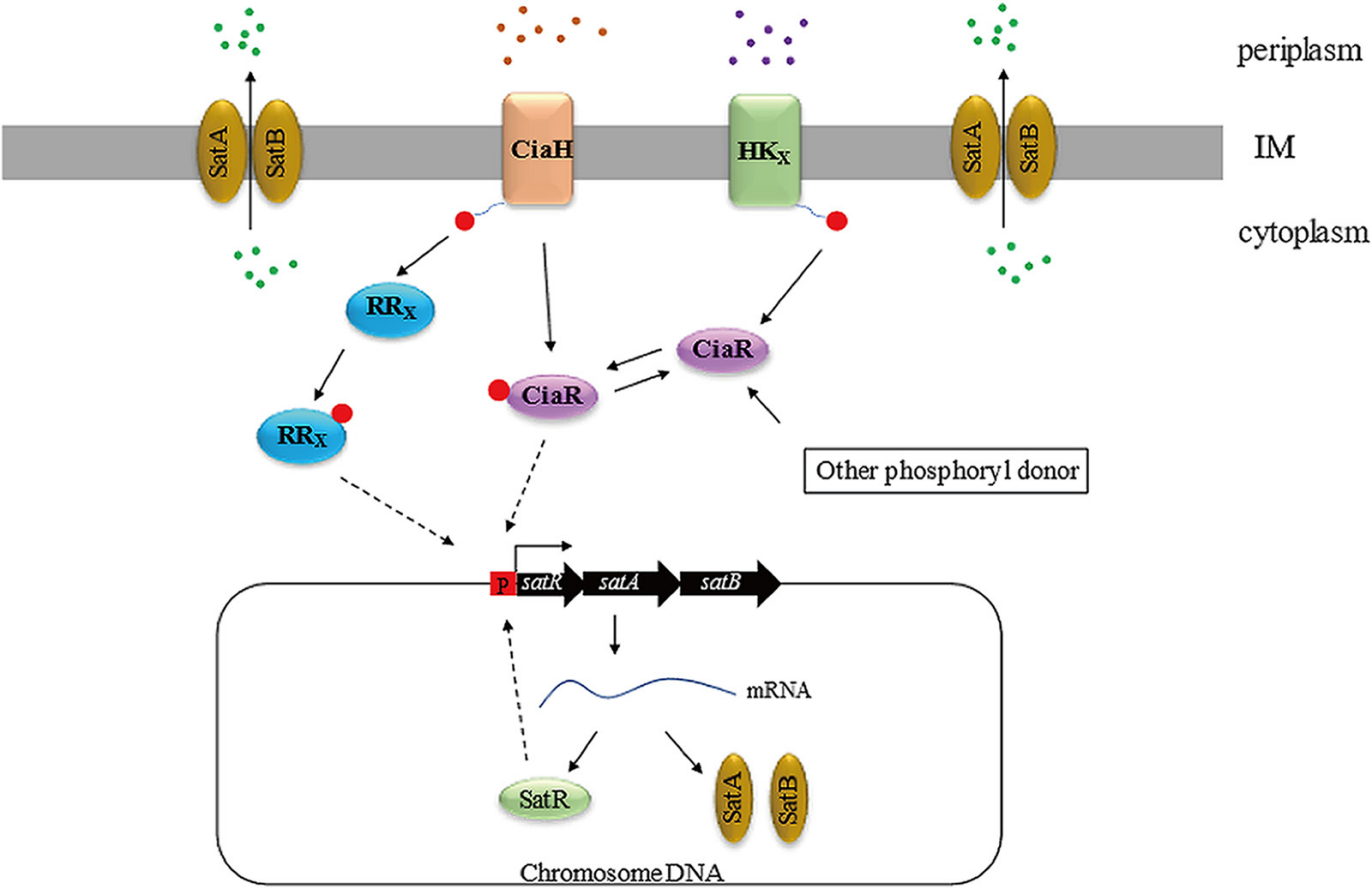
A model illustrating the regulation of SatAB expression by CiaRH and SatR in the WT. CiaRH is related to FQ resistance, and its deletion contributes to increased FQ resistance by enhancing drug discharge. In the presence of CiaR and CiaH, the addition of fluoroquinolones affects DNA replication by inhibiting gyrase or topoisomerase IV. An unknown signal released by cells is transmitted to the outside of the cell and then sensed by CiaH, which influences feedback control of the CiaRH system by an unknown mechanism. This process results in decreased activity of the CiaRH system and, thus, releases the inhibition of *satAB*. When the inhibition process is relieved, bacterial cells are able to form more pores (efflux pump SatAB) in the cell membrane. This process eventually leads to drug efflux. In the absence of CiaH, an unknown histidine kinase (HK) or other phosphate donor provides a phosphate group for CiaR. Subsequently, phosphorylated CiaR can bind to the *satAB* promoter, repressing transcription of the *satA* and *satB* genes. In the absence of CiaR, an unknown response regulator (RR) receives the CiaH phosphate group to repress the transcription of *satAB*. In addition, SatR acts as a negative regulator of SatAB. Green dots represent drugs. Stimuli are depicted with orange and purple dots, which represent the same or different stimuli. IM, inner membrane; P, promoter; HK_X_, unknown HK; RR_X_, unknown RR.

Given the similar phenotypes of the WT and Δ*ciaR* strains, there might exist an additional *satAB* regulatory mechanism in S. suis. As reported previously, a putative MarR superfamily regulator, SatR, that functions as a repressor of SatAB has been confirmed (27). Its spontaneous mutations, consisting of premature interruptions and point mutations, produce 10- to 20-fold increases in the activity of the pump (27). Indeed, in our study, an *satR* mutation in the WT background promoted high levels of *satAB* expression, whereas *satR* overexpression in the Δ*ciaRH* strain restrained the pump’s expression level, which was accompanied by a series of corresponding phenotypic changes (Table 1; Fig. S3). Therefore, the abnormal activity of SatR probably led to the more sensitive phenotype of the Δ*ciaR* mutant than of the Δ*ciaRH* mutant. Another possibility, which was also raised by other investigators, is that there might be another response regular for CiaH to repress the expression of *satAB* (63–65). Therefore, we inferred that the pathways mediated by CiaR and CiaH might be different and that this resistance phenotype was regulated by the common pathway of CiaR and CiaH. Therefore, the simultaneous deletion of CiaR and CiaH was required to release the inhibitory effect on SatAB. Based on the above-described inferences, we proposed a model to decipher the FQ resistance mechanism, in which *satAB* expression was mediated by CiaRH and SatR (Fig. 7).

Several lines of evidence support the model. A further study shows that 15 promoters are directly controlled by response regulator CiaR by means of repeat TTTAAG motifs (66). Similar sequences with subtle changes have been found in the promoter region of Streptococcus mutans (67). We searched the motif of interest in front of the *satRAB* coding sequence as well. In addition, two 9-bp pseudopalindromic sequences have also been observed in the promoter region of *satRAB*, indicating that *satR* may directly affect the expression of *satAB* (27). A synergistic effect was not found in the *ciaRH satR* double-mutant strain (Table 1; Fig. S3); instead, it presented as more sensitive to the drugs tested. The protein-protein interaction analysis showed that a network centered on SatR was formed by several proteins, including SatA, SatB, and glycerol-3-phosphate dehydrogenase. Another network centered on glycerol-3-phosphate dehydrogenase was also formed by other proteins, mainly participating in the glycerol metabolic process, peptidoglycan biosynthesis, and the lipoteichoic acid biosynthetic process. The disruption of *satR* in the Δ*ciaRH* mutant may damage the cell wall integrity and metabolic activities of S. suis, which may increase the cells’ permeability by affecting other genes in the SatR network, leading to a more antibiotic-sensitive phenotype. We speculated that the fluctuation of SatAB levels could lead to changes in growth and metabolic activities and then to different responses to antibiotics (20).

It is desirable to circumvent drug efflux pumps to enhance the activity of their corresponding antibiotics. Efflux pump inhibitors (EPIs), as promising therapeutic adjuvants, are able to interfere with the function of the pumps and, thus, restore the activity of currently existing antibiotics. To date, many strategies have been proposed in order to achieve the inhibition of efflux pumps, including (i) developing new antibiotics or modifying existing antibiotics in order for them not to be efflux pump substrates, (ii) interfering with the binding of substrates to the multidrug binding sites of pumps, (iii) blocking the functional assembly of efflux pumps, (iv) destroying the energy mechanisms driving efflux pumps, and (v) reducing the expression levels of efflux pumps by influencing the genes encoding the pumps or the regulators functioning in the expression of the efflux pumps (68). Many EPIs function based on the different strategies mentioned above, such as carbonyl cyanide *m*-chlorophenylhydrazone (CCCP) (69, 70) and verapamil (71). According to the last category, interference with the regulation of *satAB* by CiaRH may be used to downregulate the expression of the SatAB efflux pump and rejuvenate fluoroquinolone antibiotic activity. Thus, our work may provide a theoretical basis for the discovery of resistance inhibitors.

In summary, we identified a new regulatory mechanism of FQ resistance. The above-described results clearly demonstrated that CiaRH could negatively regulate the expression of the efflux pump SatAB in S. suis. This mechanism may allow the *ciaRH* mutant and even FQ-resistant clinical isolates with *satAB* overexpression to resist environmental antibiotic stimuli, thereby escaping drug killing and leading to bacterial pathogenicity.

## MATERIALS AND METHODS

### Bacterial strains, growth conditions, plasmids, primers, and antibiotics.

The bacterial strains, plasmids, and primers used in this study are presented in Tables S3 and S4. S. suis strain SC19 (wild-type strain) and its isogenic derivatives were routinely cultured in tryptic soy broth (TSB; Difco Laboratories, Detroit, MI, USA) liquid medium or tryptic soy agar (TSA; Difco Laboratories) solid medium with 10% newborn bovine serum at 37°C. E. coli strain DH5α was grown in a shaking culture in Luria-Bertani (LB) broth or a static culture on LB agar (Haibo, Qingdao, China). Spectinomycin was used at 50 mg/L and 100 mg/L for E. coli and S. suis, respectively. Norfloxacin and ciprofloxacin were purchased from Yuanye (Shanghai, China).

### Construction of the deletion mutants and functional complementation.

Chromosomal DNA from the wild-type strain (WT strain) was used as the PCR template for the construction of all plasmids. All plasmids were isolated from E. coli DH5α and transformed into electrocompetent WT cells. DNA constructs were verified by PCR and sequencing prior to application. Briefly, mutational inactivation of the target genes was achieved by homologous recombination using PCR constructs consisting of fragments of the surrounding flanking genes. For the *ciaRH satAB* double-mutant strain, the upstream and downstream regions of the *satAB* genes were PCR amplified using the following two pairs of primers: *satAB*-L1 and *satAB*-L2 and *satAB*-R1 and *satAB*-R2. The PCR products were then fused by overlap PCR using the *satAB*-L1 and *satAB*-R2 primers and subjected to digestion with restriction enzymes and ligation into similarly digested pSET4s. The recombinant vector was transformed into chemically competent E. coli DH5α cells. The correct plasmid was isolated and mobilized into the Δ*ciaRH* mutant. The same procedures used for the double mutant were followed to create other mutants and complemented strains. The SatR overexpression strain ΔciaRHCsatR was conducted by introducing the satR gene into ΔciaRH mutant. The mutants and complemented strains created for this study were selected using 100 μg/mL spectinomycin.

### Susceptibility testing.

The disc diffusion and MIC assays were conducted according to the Clinical and Laboratory Standards Institute (CLSI) guidelines (72). Briefly, strains were cultured to the exponential phase (optical density at 600 nm [OD_600_] of 0.6). The bacteria were precipitated and suspended to a 0.5-McFarland standard in a phosphate-buffered saline (PBS) solution. The suspension was inoculated onto solid medium and cultured at 37°C for 20 h. The diameters of the inhibition zones were recorded. For MIC testing, strains were added to media containing different concentrations of antibiotics at 37°C for 24 h. Parallel tests were performed in the presence of the inhibitors, reserpine and sodium orthovanadate. The lowest concentration of drugs exhibiting no visible growth was regarded as the MIC.

### Growth curves.

The stress tolerance of each strain was determined using various sub-MICs of norfloxacin or ciprofloxacin. Overnight cultures were subcultured into fresh medium with antibiotics at sub-MICs. The control groups were cultured without antibiotics. Growth curves were measured at 37°C using the Bioscreen C instrument (Oy Growth Curves Ab Ltd.).

### Liquid chromatography-tandem mass spectrometry (LC-MS/MS).

Overnight cultures of the WT and Δ*ciaRH* strains were subcultured into fresh medium and grown to the exponential phase (OD_600_ of 0.6). The bacteria were harvested by centrifugation. WT and Δ*ciaRH* pellets were lysed in SDT (4% SDS, 1 mM dithiothreitol [DTT], 100 mM Tris-HCl, pH 7.6) buffer and then used for protein extraction. Protein concentrations were measured with the bicinchoninic acid (BCA) protein assay kit (Bio-Rad, USA). Protein digestion by trypsin was performed according to the filter-aided sample preparation (FASP) procedure (73). The digested peptides of each sample were desalted on C_18_ cartridges (Empore solid-phase extraction [SPE] cartridges, C_18_ SD [standard density], 7-mm bed inner diameter, 3-mL volume; Sigma), concentrated by vacuum centrifugation, and reconstituted in 40 μL of 0.1% (vol/vol) formic acid. The peptide mixture obtained by trypsin digestion was labeled using TMT reagent according to the manufacturer’s instructions (Thermo Scientific).

The labeled peptides from each group were mixed equally and then fractionated using a high-pH reversed-phase peptide fractionation kit (Thermo Scientific). First, acetonitrile and 0.1% trifluoroacetic acid (TFA) were used for column equilibration. Then, the dried peptide mixture was loaded onto a spin column and bound to the hydrophobic resin, which was subsequently desalted by washing the column with water by low-speed centrifugation. Bound peptides were eluted into different fractions with a gradient of acetonitrile in a volatile high-pH elution solution. The collected fractions were desalted on C_18_ cartridges (Empore SPE cartridges, C_18_ SD, 7-mm bed inner diameter, 3-mL volume; Sigma) and concentrated by vacuum centrifugation.

LC-MS/MS analysis was conducted on a Q Exactive mass spectrometer (Thermo Scientific) coupled to an Easy-nLC injector (Proxeon Biosystems, now Thermo Fisher Scientific) for 60/90 min. The peptides were loaded onto a reverse-phase trap column (Acclaim PepMap100, 100 μm by 2 cm, NanoViper C_18_; Thermo Scientific) connected to a C_18_ reversed-phase analytical column (Easy-Spray column, 10 cm long, 75-μm inner diameter, 3-μm resin; Thermo Scientific) in buffer A (0.1% formic acid) and separated with a linear gradient of buffer B (84% acetonitrile and 0.1% formic acid) at a flow rate of 300 nl/min, controlled by IntelliFlow technology. The mass spectrometer was operated in positive-ion mode. MS data were acquired using a data-dependent top-10 method dynamically choosing the most abundant precursor ions from the survey scan (300 to 1,800 *m/z*) for high-energy collisional dissociation (HCD) fragmentation. The automatic gain control (AGC) target was set to 3e6, and the maximum injection time to 10 ms. The dynamic exclusion duration was 40.0 s. Survey scans were acquired at a resolution of 70,000 at *m/z* 200, the resolution for HCD spectra was set to 17,500 at *m/z* 200, and the isolation width was 2 *m/z*. The normalized collision energy was 30 eV, and the underfill ratio, which specifies the minimum percentage of the target value likely to be reached at the maximum fill time, was defined as 0.1%. The instrument was run with peptide recognition mode enabled.

The identification and quantitation analysis of MS raw data were performed using the MASCOT engine (version 2.2; Matrix Science, London, UK) and Proteome Discoverer 1.4 software. The NCBI BLAST+ client software (ncbi-blast-2.2.28+-win32 executable file) and InterProScan were used to locally search the differentially expressed protein (DEP) sequences to find homologous sequences. Gene ontology (GO) terms (http://geneontology.org/) were mapped, and sequences were annotated using the software program Blast2GO. The protein subcellular localization and domain signatures were annotated by using CELLO (subCELlular LOcalization predictor; http://cello.life.nctu.edu.tw/) and InterProScan software, respectively. The selected DEPs were blasted from the online Kyoto Encyclopedia of Genes and Genomes (KEGG) database (https://www.genome.jp/kegg/). KEGG analysis retrieved their KEGG orthology identifications, and DEPs were subsequently mapped to pathways in KEGG. GO and KEGG pathway enrichment analyses were applied based on Fisher’s exact test, considering the whole quantified proteins as the background data set. Benjamini-Hochberg correction for multiple testing was further applied to adjust the derived *P* values.

### RNA isolation and quantitative real-time PCR (qRT-PCR).

WT and Δ*ciaRH* strains in the mid-exponential phase (OD_600_ of 0.6) were divided into five equal parts, which were supplemented with growth medium containing different concentrations of norfloxacin. The WT strain in growth medium with no norfloxacin served as the control. These suspensions were further cultured for 10 min and then collected for RNA extraction. The Δ*satR*, Δ*ciaRH* Δ*satR*, and Δ*ciaRH* C*satR* strains were grown to the mid-exponential phase and then collected. The WT strain in the exponential phase was used as the control. RNA extraction was in accordance with the bacteria total RNA isolation kit instructions (Sangon Biotech [Shanghai] Co., Ltd.). Agarose gel electrophoresis was used to examine the RNA integrity. The RNA was subjected to cDNA synthesis using a Hiscript III RT supermix for qPCR kit (+gDNA wiper) (Vazyme). Quantitative PCR was performed using a SYBR green master mix on the ABI ViiA 6 system. The level of gene expression was normalized to that of 16S rRNA and analyzed employing the cycle threshold (2^−ΔΔ^*^CT^*) method.

### Norfloxacin accumulation and efflux detections.

Antibiotic accumulation in and efflux from the cells of S. suis strains in the presence of norfloxacin were determined using a microplate reader (Tecan). Bacterial strains were cultured overnight and diluted 100-fold into fresh medium. For the accumulation assay, the strains were incubated with norfloxacin (8 μg/mL) when grown to mid-log phase (OD_600_ of 0.6). Samples were removed at 30 min. The cells were harvested by centrifugation (2,300 × *g* at 4°C for 5 min) and washed with Tris-HCl (pH 7.0) following lysis via sonication. The supernatant obtained was used to detect the fluorescence. For the efflux assay, cells were centrifuged, washed with Tris-HCl (pH 7.0), and then resuspended in Tris-HCl (pH 3.0). The strains were incubated with norfloxacin (512 μg/mL) at 37°C for 5 min. Glucose (25 mM) was added before measurement. Norfloxacin efflux was measured by a microplate reader. The excitation and emission wavelengths were 277 nm and 448 nm, respectively.

### Accumulation of ethidium bromide.

The activity of the efflux pump SatAB in the *ciaRH* mutant, grown to mid-log phase (OD_600_ of 0.6) in TSB medium, was compared with its activities in the WT and *ciaRH satAB* double-mutant strains by monitoring the uptake of ethidium bromide to a final concentration of 10 μM. After 10 min, measurements were taken at excitation and emission wavelengths of 485 nm and 535 nm, respectively, with a microplate reader.

### Capping-RACE.

A capping-RACE assay was performed as previously described (74). The total RNA from WT was prepared as described above and treated with DNase I. Capping of 10 μg RNA was completed using the vaccinia virus capping enzyme (VCE) system (NEB). cDNA was subsequently synthesized with SuperScript III reverse transcriptase (Invitrogen), where a template-switching oligonucleotide (TSO) containing a 3′-end poly(rG) tail was used to form base pairs with the extra cytosine residues of cDNA. Nested PCR was performed using the outer primer and GSP1 as the first-round PCR, followed by the second-round PCR with the inner primer and GSP2. The PCR products were purified using a Cycle-Pure kit (Omega) and cloned into the pMD18-T vector (TaKaRa). Sequences were obtained by sequencing.

### Identification of the cotranscription of *satRAB*.

The RNA isolation and cDNA synthesis of WT were the same as described above. PCRs from *satR* to *satA*, from *satA* to *satB*, and from *satR* to *satB* were performed using cDNA, total RNA, and genomic DNA originating from WT samples. A negative control was implemented with diethyl pyrocarbonate (DEPC)-treated water instead of nucleic acid template.

### Electrophoretic mobility shift assays (EMSAs).

The DNA fragments of full-length CiaR and SatR from the S. suis WT strain were amplified by PCR. Then, the fragments were subcloned into the pET-30a(+) plasmid with a 6× His tag, forming pET-30a(+)–CiaR and pET-30a(+)–SatR. The recombinant plasmids were confirmed by sequencing. E. coli BL21(DE3) was used for heterologous expression, which was induced with 1 mM isopropyl-1-thio-d-glactopyranoside at 37°C for 4 h. Then, the E. coli cells were centrifuged, resuspended, and next destroyed by using a low-temperature ultrahigh-pressure continuous-flow homogenizer (JNBIO, China). The resulting suspensions were further centrifuged to remove cell debris. Both proteins were purified by Ni-nitrilotriacetic acid (NTA) resin affinity chromatography. Purified CiaR and SatR proteins were dialyzed in buffer containing 25 mM Tris, pH 7.4, 150 mM NaCl, 5% glycerol, 0.5 mM EDTA, and 1 mM DTT. Protein purity was judged by SDS-PAGE. Among the proteins, CiaR was phosphorylated for 10 min with acetyl phosphate before use (Sigma, USA) (75).

Complementary oligonucleotides and primers for probes are presented in Table S3. The *ciaR* promoter region, which was used as a positive probe for the CiaR binding reaction, was amplified using primers EMSApos-*ciaR*-F and -R. The other probes were formed by self-annealing of complementary oligonucleotides. These probes were end labeled with biotinylated ribonucleotides using a biotin 3′-end DNA labeling kit (Beyotime). The EMSA was performed according to the manufacturer’s instructions (EMSA/gel-shift kit; Beyotime). The binding reactions of CiaR or SatR protein with the labeled probes were performed in an EMSA binding buffer for 20 min at room temperature. The mixture was separated in a 4% native polyacrylamide gel (1 mL 10× TBE buffer [0.9 mol/L Tris-borate, 0.02 mol/L EDTA], 16.2 mL pure water, 2 mL 39:1 acrylamide/bisacrylamide (40%, wt/vol), 0.15 mL 10% ammonium persulfate, 0.01 mL TEMED [*N*,*N*,*N*′,*N*′-tetramethylethylenediamine], and 0.625 mL 80% glycerol) at 70 V and transferred to a positively charged nylon membrane at 100 V for 40 min, followed by UV cross-linking and incubation with a horseradish peroxidase (HRP)-conjugated antibody. Finally, the probes were detected with a ChemoDoc touch imaging system (Bio-Rad).

### Affinity measurements between SatR, CiaR, and the promoter region of *satAB*.

The interactions between both proteins and the *satAB* promoter region were determined using streptavidin (SA) biosensors in the Octet RED96 system (ForteBio, Inc., Menlo Park, CA, USA). First, the DNA probes were biotinylated and diluted 200-fold to 50 nM. Then, the proteins at 1 mg/mL (CiaR and SatR) underwent two doubling dilutions in PBS. The equipment was turned on for 30 min before use to prewet the sensors (streptavidin) in phosphate-buffered saline with Tween 20 (PBST). The program was operated in the following order: baseline (60 s), loading (300 s), baseline 2 (300 s), association (300 s), and dissociation (600 s). The data were analyzed using the ForteBio Octet analysis software (ForteBio, Menlo Park, CA).

### Statistical analysis.

GraphPad Prism 8 was used for data analysis. DEPs were defined with fold changes of >1.20 or <0.83 in comparison to the expression in the wild-type strain and a *P* value of <0.05. All data are shown as mean values ± standard deviations. Variances in data between strains were analyzed for statistical significance using Student’s *t* test. Data were recognized as significantly different with a *P* value of <0.05.

### Data availability.

The mass spectrometry proteomics data are available via ProteomeXchange with identifier PXD033553.
