# Supplementary material for: Role of the Two-Component System CiaRH in the Regulation of Efflux Pump SatAB and Its Correlation with Fluoroquinolone Susceptibility
Source: Microbiol Spectr. 2022 May 31;10(3):e00417-22. doi: 10.1128/spectrum.00417-22 (PMC9241815; doi:10.1128/spectrum.00417-22)
Supplement: SUPPLEMENTAL FILE 1 — Supplemental material. Download spectrum.00417-22-s0001.pdf, PDF file, 0.8 MB [file spectrum.00417-22-s0001.pdf]

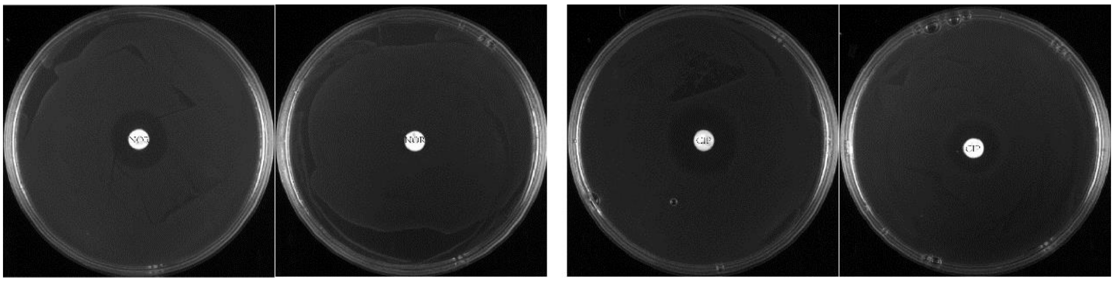

WT	∧*ciaRH*	WT	∧*ciaRH*

Figure S1 Diffusion antibiogram of strains wild type (WT) strain and Δ*ciaRH*. Inhibition zones are smaller for Δ*ciaRH* than for parental strain wild type, proving the implication of CiaRH-mediated fluoroquinolone resistance. CIP, ciprofloxacin; NOR, norfloxacin.

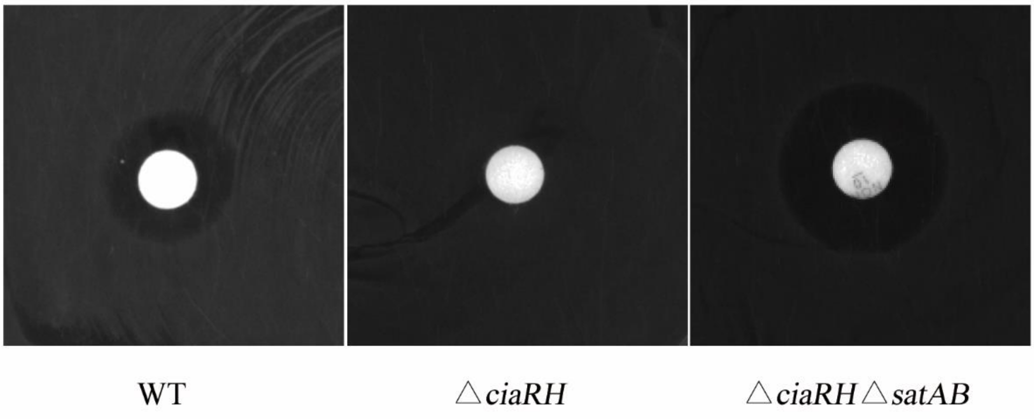

Figure S2 Diffusion antibiogram of strains wild type (WT) strain, Δ*ciaRH* and Δ*ciaRH*Δ*satAB*. Inhibition zones are bigger for Δ*ciaRH*Δ*satAB* than Δ*ciaRH*, proving the implication of *satAB*-mediated fluoroquinolone resistance. NOR, norfloxacin.

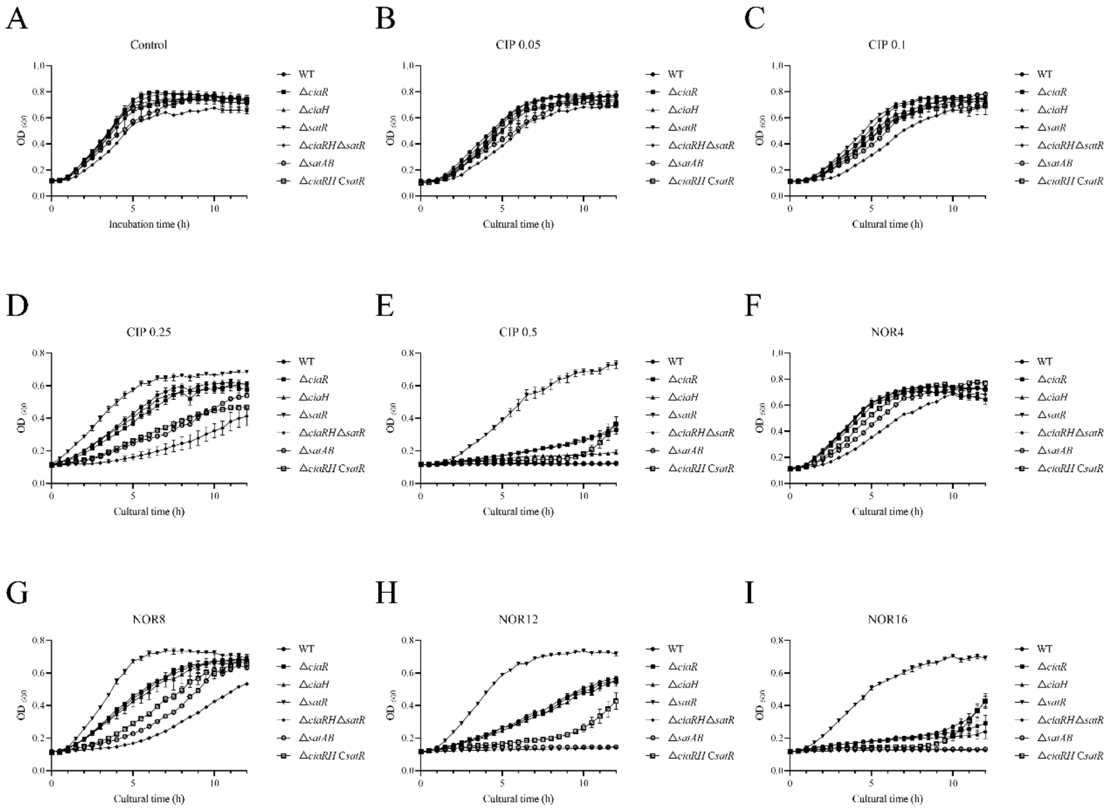

Figure S3 Growth curve of related gene deletion strains in liquid medium. Growth curves of the wild type (WT), Δ*ciaR*, Δ*ciaH*, Δ*satR*, Δ*ciaRH*Δ*satR*, Δ*satAB* and Δ*ciaRH* C*satR* strains in the absence (A) and presence of 0.051μg/mL(B), 0.1μg/mL (C), 0.25μg/mL (D), and 0.5μg/mL (E) CIP, or 4μg/mL (F), 8μg/mL (G), 12μg/mL (H), and 16μg/mL (I) NOR. CIP, ciprofloxacin; NOR, norfloxacin. The data in the graphs are the means and SD from three wells.

Table S1 Information of all identified proteins.

Protein IDs	ΔCiaRH/WT	protein_name
A0A075SM39	4.398316	Multidrug ABC transporter permease
A0A116KEC8	3.610729	ABC transporter ATP-binding protein
A0A0Z8K183	3.381022	Translation initiation factor 1
A0A2I5KP51	2.945995	Cytosine-specific methyltransferase
A0A3R8RG27	2.81065	UDP-N-acetylmuramoyl-tripeptide--D-alanyl-D-alanine ligase
A0A123TPT9	2.636976	DDE transposase
A0A426T3G1	2.465703	Phage tail protein
A0A4T2GRG8	2.447531	ATPase
Q204C2	2.425941	IgG binding protein
D5AHE9	2.423117	DegV
A0A123SUD5	2.381728	Multiple sugar transport system substrate-binding protein
A0A0Z8XUH7	2.362783	Uncharacterized protein
D5AIW5	2.305527	Transcriptional antiterminator bglG
A0A0Z8N6A5	2.294889	Putative oligopeptidase
A0A123TF73	2.226447	Multidrug ABC transporter ATPase and permease
A0A0Z8D4F3	2.170209	NH(3)-dependent NAD(+) synthetase
A0A116P1S9	2.157423	Dihydroxyacetone kinase
D5AJB3	2.133833	Thiolase
A0A0Z8GGW1	2.124094	Pyridine nucleotide-disulfide oxidoreductase
A0A426TB70	2.109284	Phospho-2-dehydro-3-deoxyheptonate aldolase
A0A3R8N2V7	2.090135	Cystathionine gamma-synthase
A0A123URJ9	2.082671	Acetyl-CoA acetyltransferase
A0A1X9I2M8	2.07476	Histidine kinase
G7SM50	2.054408	-
A0A0Z8C9D8	2.042538	Tyrosine recombinase XerD-like
A0A0Z8D6U1	1.970883	Transporter
A0A075SEI5	1.968108	Acetate--CoA ligase
G7SKN7	1.939174	-
A0A0Z8EC68	1.93346	Extracellular serine protease
A0A0Z8H0L5	1.91912	Xaa-Pro aminopeptidase
D5AJR0	1.917362	Biotin carboxylase
A0A0Z8R5Y3	1.913502	3-hydroxy-3-methylglutaryl CoA synthase
A0A0Z8KLR5	1.911084	Biotin carboxylase
D5AJI4	1.9095	rRNA methyltransferase
A0A0N0DP09	1.892029	ABC transporter
A0A4T2H419	1.878626	Enoyl-CoA hydratase
A0A3R8RA20	1.866085	Hypoxanthine phosphoribosyltransferase
A4W3M3	1.864135	3-hydroxyacyl-[acyl-carrier-protein] dehydratase FabZ
D5AEQ8	1.862578	DNA repair protein RadA
A0A2Z4PHI0	1.853302	GRAM_POS_ANCHORING domain-containing protein
D5AJ07	1.849596	FAD:protein FMN transferase
D5AGU8	1.846517	Aminotransferase

A0A126UMQ5	1.840471	dTDP-4-dehydrorhamnose reductase
A0A0Z8J0U4	1.83742	DegV family protein
A0A0Z8F247	1.826148	Serotype determinant, transmembrane phosphoglyceroltransferase
A0A116M3C7	1.823979	Cysteine synthase
G7SHM0	1.808092	Acetyl-coenzyme A carboxylase carboxyl transferase subunit alpha
A4VWD1	1.806448	tRNA modification GTPase MnmE
A0A116NQ34	1.803246	Phosphate import ATP-binding protein PstB
A0A4V4RXD1	1.797934	Lipoate--protein ligase
A0A426T4Y1	1.796885	Endopeptidase
D5AI55	1.788071	Uncharacterized protein
A4VX00	1.775669	NH(3)-dependent NAD(+) synthetase
D5AJR3	1.774396	3-oxoacyl-[acyl-carrier-protein] synthase 2
D5AKA0	1.765758	Ribulose-phosphate 3-epimerase
A0A0Z8GPQ8	1.765334	Lipoate--protein ligase
A4W0U7	1.764178	4-hydroxy-tetrahydrodipicolinate reductase
A0A4V4RWH5	1.75494	LytR family transcriptional regulator
A0A4T2H574	1.752915	60 kDa chaperonin
A0A0Z8G3H4	1.749703	Response regulator
D5AIW7	1.749614	Alpha-acetolactate decarboxylase
A0A0M9FD88	1.746159	1,2-diacylglycerol 3-glucosyltransferase
A0A0Z8E850	1.737095	Aminotransferase
A4VSA7	1.736202	Phosphate acyltransferase
A0A116LCY7	1.730381	Riboflavin transporter
A0A2I5KGF1	1.729898	Uncharacterized protein
A0A426G2C9	1.729056	LPXTG cell wall anchor domain-containing protein
A0A0N1J3P7	1.727487	NAD kinase
A0A0Z8GJX2	1.725344	Uncharacterized conserved small protein
A4W0T4	1.720383	GMP reductase
A0A0M9FJ69	1.71796	Uncharacterized protein
A0A0Z8KT40	1.717139	DNA polymerase III subunits gamma and tau
A0A075SNP6	1.715755	Dihydrofolate synthase
A0A2K1T1V8	1.715741	Cyclase
A0A0Z8ILU3	1.706954	D-alanyl-D-alanine carboxypeptidase
A0A0M9FLV5	1.700733	Ribosomal RNA small subunit methyltransferase I
A0A0Z8D7J4	1.699048	Septum formation initiator
A0A4T2GUI9	1.697789	Phosphate import ATP-binding protein PstB
D5AJD9	1.69767	Probable oligoendopeptidase F
A0A0Z8L402	1.697667	3-oxoacyl-[acyl-carrier-protein] synthase 2
A0A0Z8C6U0	1.690817	FMN-binding protein
A0A116JHI1	1.687491	N-acetylglucosamine-6-phosphate deacetylase
A0A123TKU8	1.672828	tRNA modification GTPase MnmE

A4VW37	1.670918	Response regulator consisting of a CheY-like receiver domain and a winged-helix DNA-binding domain
A0A3R8N1U9	1.668091	Peptidase T
A0A116LBB9	1.664684	Putative oligopeptidase
A0A0Z8N5X9	1.661609	NH(3)-dependent NAD(+) synthetase
A0A116MK19	1.659268	ArcT
A0A0Z8F539	1.658302	Aspartate-semialdehyde dehydrogenase
A0A0Z8G4T9	1.658162	Acetyl-coenzyme A carboxylase carboxyl transferase subunit beta
A0A2I5KPE5	1.657838	Resolvase
A0A4T2GXW8	1.657659	FAD-containing oxidoreductase
A0A0Z8T3U4	1.657052	Pyridine nucleotide-disulfide oxidoreductase
A0A116M9S6	1.648824	Pyridine nucleotide-disulfide oxidoreductase
A0A4T2HGM8	1.648351	3-dehydroquinate synthase
A0A0Z8H190	1.647046	Ribose-phosphate pyrophosphokinase
A0A0Z8B6H6	1.644648	N-acetyl-beta-hexosaminidase
A0A0Z8IU19	1.642759	Sugar ABC transporter periplasmic protein
A0A0Z8P5F8	1.64138	O-acetylserine lyase
A0A2Z4PJI2	1.64105	Uncharacterized protein
A0A126UN30	1.638834	ABC transporter
A0A4T2HEW5	1.638803	Alpha-galactosidase
A0A0Z8EPH3	1.638752	Exopolysaccharide biosynthesis protein, glycosyltransferase
A0A0Z8D745	1.635056	Dihydrofolate reductase
D5AI23	1.633733	ATP synthase subunit beta
A0A345S365	1.630812	M3 family oligoendopeptidase
D5AJR2	1.629316	Biotin carboxyl carrier protein of acetyl-CoA carboxylase
A0A2S2F5Q5	1.625546	Cystathionine gamma-synthase
A0A0Z8IWG1	1.611235	Xaa-Pro aminopeptidase
A0A1X9I392	1.610949	Uncharacterized protein
A0A0Z8HJ65	1.607274	Membrane carboxypeptidase
A0A0Z8B6J1	1.606652	S-adenosylmethionine:tRNA ribosyltransferase-isomerase
A0A0Z8J805	1.605135	ATPase
D5AJA0	1.60241	DNA polymerase III subunit gamma/tau
A0A0Z8GJZ1	1.597312	Phosphomevalonate kinase
A0A0Z8IRV9	1.596936	Cysteine aminopeptidase C
D5AJU3	1.596869	Lipoate--protein ligase
D5AJW5	1.596603	Cell envelope-related transcriptional attenuator
G7SLI8	1.596225	-
A0A0M9FGR5	1.594088	Glycine--tRNA ligase alpha subunit
A0A075SJV9	1.593668	Pseudouridine synthase
A0A116PJ44	1.591791	ATPase
D5AHS6	1.588487	Phosphate acetyltransferase
A0A075SCV4	1.587076	Chorismate synthase
D5AK56	1.587007	Aminopeptidase PepS

A0A0N0DM93	1.585377	PucR family transcriptional regulator
A0A0Z8F1P8	1.584966	Ribosomal protein L11 methyltransferase
A0A3R8MWS5	1.582098	Glycogen synthase
A0A0Z8MD08	1.581151	Malonyl CoA-acyl carrier protein transacylase
A0A142UMQ8	1.579148	CRISPR-associated endonuclease Cas1
D5AHJ3	1.577061	Phosphoglucomutase
A0A0N0VCE3	1.571902	Phenylalanine--tRNA ligase alpha subunit
A0A426TDP3	1.569322	Glyceraldehyde-3-phosphate dehydrogenase
A0A0Z8QBX4	1.568842	Integral membrane protein
A0A075SF72	1.563275	ADP-ribose pyrophosphatase
A0A0Z8L1Q0	1.563119	ABC transporter ATP-binding protein
A0A426TIZ7	1.561984	DEAD/DEAH box helicase
A0A0Z8JE90	1.561511	D-alanine--D-alanyl carrier protein ligase
A0A0H3N5V1	1.557294	Thioredoxin reductase
A0A0N0DND8	1.551296	Probable manganese-dependent inorganic pyrophosphatase
A4VX55	1.549328	DNA replication protein
A0A0Z8EDH5	1.547582	TrmH family RNA methyltransferase
A0A0Z8F860	1.546884	ABC transporter ATP-binding protein
A0A075SKJ3	1.546236	Isoprenyl transferase
A0A142UR48	1.541969	dITP/XTP pyrophosphatase
A0A075SFL4	1.541066	Glycosyl transferase family 1
A0A2K1T0M4	1.53737	UDP-N-acetylmuramoyl-tripeptide--D-alanyl-D-alanine ligase
A4W440	1.537313	Adenylosuccinate synthetase
A0A0Z8LQ97	1.536073	Lipid kinase
A0A0N0VB36	1.53459	Diacylglycerol kinase
A0A0Z8HHI8	1.534427	DegV family protein
A0A0Z8CIY9	1.533027	Transcriptional regulator
A0A0Z8AWD9	1.530563	ABC transporter ATP-binding protein
A0A0Z8CX51	1.530502	Phosphatase/phosphohexomutase
A0A0Z8FM12	1.528199	60 kDa chaperonin
A0A0Z8JKH7	1.525664	Ribonuclease HII
A0A0Z8IFJ9	1.523548	Putative phosphate ABC transporter, extracellular phosphate-binding lipoprotein
A0A168YV45	1.523417	Oligoendopeptidase F
A0A0Z8AXE4	1.523229	Thymidylate synthase
A0A4T2HF59	1.521635	Uncharacterized protein
A0A116L7C2	1.521173	Cysteine aminopeptidase C
A0A123U461	1.520681	Deoxyuridine 5'-triphosphate nucleotidohydrolase
A0A0Z8G3B1	1.519177	O-acetylserine lyase
D5AHE6	1.519112	Tagatose-6-phosphate kinase
A0A426TCE4	1.518715	FAD:protein FMN transferase
D5AGZ6	1.51814	Putative DNA polymerase III, delta subunit
A4W4N7	1.517057	tRNA-specific 2-thiouridylase MnmA
A0A0Z8FB72	1.513285	D-alanine--D-alanine ligase

A0A116MN37	1.512487	Pyruvate/2-oxoglutarate dehydrogenase complex, dehydrogenase (E1) component, alpha subunit
D5AIW4	1.512106	Sugar-specific permease, EIIA 1 domain:PTS system, beta-glucoside-specific IIABC component
A0A0Z8C1R1	1.512028	3'-exo-deoxyribonuclease
D5AH93	1.510198	DUF1232 domain-containing protein
A0A0Z8GNN7	1.509092	Pantothenate kinase
A0A4T2H2N3	1.508811	YSIRK-type signal peptide-containing protein
A0A4T2GJY3	1.508551	Diaminopimelate decarboxylase
A0A0M9FK23	1.508366	Peptidase
A4VWD9	1.508125	Dipeptidase
D5AIF5	1.507861	Short-chain dehydrogenase/reductase SDR
A0A123SSS7	1.506033	GMP synthase [glutamine-hydrolyzing]
A0A4T2GQK6	1.504109	Uncharacterized protein
A0A0Z8CBW1	1.501215	Molybdenum ABC transporter ATPase
A0A0Z8W3R4	1.499691	CpsY
A0A4T2GYQ4	1.499342	Glutathione-disulfide reductase
D5AIC3	1.498931	Glycosyl transferase, family 2
A0A116R1H6	1.498729	Esterase
A0A0Z8K7B2	1.497478	Glutathione biosynthesis bifunctional protein GshAB
A0A0Z8MMW2	1.497379	Type I restriction-modification system methylation subunit
A0A0N0DPB0	1.496598	Membrane protein
Q3Y455	1.495691	Glyceraldehyde-3-phosphate dehydrogenase
A0A0Z8B8G2	1.49562	Phage protein
A0A123T7E1	1.493534	S4 domain-containing protein YaaA
A0A0M9FI43	1.493147	L-lactate dehydrogenase
A0A0M9FIF1	1.491866	N-acetyldiaminopimelate deacetylase
A0A4T2GR93	1.488048	Insulinase family protein
A0A0Z8CGL9	1.487043	tRNA-specific 2-thiouridylase MnmA
A0A0Z8KDX0	1.486461	Lipase
A0A0M9FI13	1.486159	Primosomal protein DnaI
D5AJM8	1.485982	NAD+ binding protein: Trk transporter
A0A3R8R6V3	1.484749	DUF3991 domain-containing protein
D5AIU9	1.484413	Putative UDP-N-acetylmuramyl tripeptide synthetase MurC
A0A0Z8H8J9	1.482117	Holliday junction ATP-dependent DNA helicase RuvB
A0A0Z8ILS5	1.481023	Phosphate regulon transcriptiona lregulatory protein PhoB (SphR)
A0A0Z8E3X9	1.479777	HTH DNA-binding protein
A0A0Z8DIE6	1.479323	Beta-glucosidase/6-phospho-beta-glucosidase/beta-galactosidase
A0A0Z8R1Q3	1.478127	Uncharacterized protein

D5AH25	1.477489	FAD-dependent pyridine nucleotide-disulfide oxidoreductase:Pyridine nucleotide-disulfide oxidoreductase dimerization region
A0A2I5KEG5	1.477255	ATP synthase gamma chain
A0A0M9FIU5	1.477011	Acyl-phosphate glycerol 3-phosphate acyltransferase
A0A0Z8HBH4	1.476811	3-oxoacyl-[acyl-carrier-protein] synthase 3
A0A116KMM3	1.474132	Copper-transporting ATPase
A0A0Z8NN76	1.471119	Tn5252 Orf26
A0A0N0DQ50	1.470573	DNA repair protein
D5AFV5	1.470095	Zinc-containing alcohol dehydrogenase
A0A0Z8DNT7	1.468973	Type I secretion system ATPase
A0A123UAQ3	1.468735	Energy-coupling factor transporter ATP-binding protein EcfA
A0A0Z8G7P7	1.466922	Cysteine desulfurase
A0A160JSA3	1.466701	Uncharacterized protein
A0A0Z8CWF1	1.466574	Fructose-2,6-bisphosphatase
D5AH19	1.466054	Putative phosphomannomutase
A0A3R8SIG4	1.464607	Glycine--tRNA ligase alpha subunit
A0A0Z8DG13	1.46206	Folylpolyglutamate synthetase
A0A0Z8G021	1.461361	Phospho-2-dehydro-3-deoxyheptonate aldolase
A0A116TL77	1.461282	Ribosome-binding ATPase YchF
A0A116NXD5	1.459856	3-isopropylmalate dehydrogenase
A0A0Z8J6F9	1.45965	Redox-sensing transcriptional repressor Rex
A0A0N0DN57	1.459475	Uracil-DNA glycosylase
G7SJB7	1.458731	-
A0A0Z8EVU0	1.457806	Glutamate 5-kinase
A0A0Z8I260	1.457216	Response regulator
A0A0H3MV16	1.457127	Phenylalanine--tRNA ligase alpha subunit
A0A0M9FEM3	1.456387	Tellurite resistance protein TehB
A0A3R8T9T6	1.453782	Aspartate/glutamate racemase family protein
A0A4T2H6C3	1.452377	3-oxoacyl-[acyl-carrier-protein] reductase
D5AGF3	1.452292	Peptidase U32
A0A0Z8FKA8	1.452053	Methionyl-tRNA formyltransferase
D5AFH7	1.45199	Adenine-specific DNA methylase
A0A0Z8F6W3	1.450956	Branched-chain-amino-acid aminotransferase
A0A0Z8GV01	1.450199	Cellulase M-like protein
A0A0Z8KVY1	1.449976	Protein RecA
A0A0Z8HI92	1.449405	Phosphoglycerate kinase
A0A075SEY7	1.448824	Penicillin-binding protein
A0A4T2GLV1	1.447452	Dihydrolipoamide acetyltransferase component of pyruvate dehydrogenase complex
D5AI20	1.447238	UDP-N-acetylglucosamine 1-carboxyvinyltransferase
A0A0M9FKZ7	1.446324	ATP synthase subunit b
A0A426TBT5	1.446229	Gfo/Idh/MocA family oxidoreductase
A0A4T2H9S6	1.446192	UPF0154 protein FAJ34_04775

A0A0Z8B5Q1	1.446168	Short-chain alcohol dehydrogenase
D5AJB5	1.444739	3-hydroxy-3-methylglutaryl coenzyme A reductase
A0A2K1SYQ0	1.44454	Uncharacterized protein
A0A3R8RH88	1.440922	Nucleotide-binding protein EI993_01060
A0A0H3MUD5	1.440146	3-dehydroquinate synthase
A0A116LLU0	1.439061	dTDP-glucose 4,6-dehydratase
A0A0K2E8S7	1.438553	Glucose-6-phosphate isomerase
A0A4T2HCJ5	1.437095	UTP--glucose-1-phosphate uridylyltransferase
A0A0Z8CLZ7	1.43543	HD-superfamily hydrolase
A0A3R8R212	1.435171	LPXTG cell wall anchor domain-containing protein
A0A4T2GUH8	1.43414	Elongation factor Ts
A0A0Z8KS91	1.433537	ABC transporter ATPase
A0A0Z8BMG3	1.43175	Metalloendopeptidase
A0A0M9FN86	1.431617	Phosphate starvation-inducible protein PhoH
A0A116NJJ5	1.431005	HAD superfamily hydrolase
A0A0Z8DRF6	1.430106	Sucrose-6-phosphate hydrolase
A0A116L6G9	1.429243	TrmH family RNA methyltransferase
A0A2I5N3Y8	1.423054	Ribonuclease HII
A0A3R8TA40	1.422875	Pyrimidine-nucleoside phosphorylase
A0A0Z8FNM0	1.42209	Uncharacterized protein
A0A116M5S6	1.42205	LPXTG cell wall surface protein
A0A160JS12	1.421069	Otitis media-associated H10
A0A0N0VCK8	1.420782	Probable cell division protein WhiA
D5AF84	1.420606	Uncharacterized protein
A0A0Z8J4A3	1.420383	Group 1 glycosyl transferase
A0A075SKB2	1.420135	Pyrroline-5-carboxylate reductase
D5AHT0	1.417997	Adenylate cyclase
A0A4V6U7I9	1.417756	UDP-N-acetylmuramoyl-tripeptide--D-alanyl-D-alanine ligase
A0A123U8M6	1.416902	UPF0176 protein ERS132441_01570
A0A0K2E8K5	1.415408	HTH DNA-binding protein
A0A0Z8H760	1.415383	DNA-binding protein
A0A0Z8T6M7	1.414176	Xaa-Pro aminopeptidase
A0A0Z8F111	1.413033	Mannose-6-phosphate isomerase
A4W1Q9	1.41246	Peptidase T
A0A0Z8IAV8	1.41226	DEAD-box ATP-dependent RNA helicase CshB
D5AIL0	1.411724	Catabolite control protein A
A0A4V0EV94	1.411687	Phosphatase
A0A0M9FKD3	1.411488	Alpha-ketoacid dehydrogenase subunit beta
D5AF09	1.410153	Putative oxidoreductase ydhF
A4VSN8	1.409885	Phosphoglycerate kinase
D5AGB5	1.409192	Uncharacterized protein
A0A1X9I458	1.408769	Uncharacterized protein
D5AI93	1.406906	Oligoendopeptidase F
D5AIG2	1.406419	Meth_synt_2 domain-containing protein

A0A426T6W7	1.406136	Deoxyuridine 5'-triphosphate nucleotidohydrolase
A0A0H3MXE2	1.405415	LemA family protein
A0A0Z8FKI5	1.40464	Cobalt ABC transporter ATPase
D5AI39	1.403626	Cof protein: HAD-superfamily hydrolase, subfamily IIB
A0A0Z8IRX1	1.402312	Branched-chain-amino-acid aminotransferase
A0A0Z8GIV3	1.402148	Cysteine sulfinate desulfinase/cysteine desulfurase
A0A0Z8F1J7	1.402137	Sugar metabolism transcriptional regulator
A0A3R8SXE9	1.401802	Tryptophan--tRNA ligase
A4VVB3	1.401119	Thymidine phosphorylase
A0A4P7WNP1	1.400472	Serine hydroxymethyltransferase
D5AGR9	1.399952	UDP-N-acetylglucosamine 2-epimerase
A0A2K1SZH0	1.398814	DeoR family transcriptional regulator
A0A4T2GL76	1.398178	YigZ family protein
A0A4V0EZH0	1.398135	UDP-N-acetylmuramoylalanine--D-glutamate ligase
A0A075SJI9	1.39722	Putative ribose-phosphate pyrophosphokinase
A0A2I5KEN5	1.396555	tRNA (Adenine-N(1))-methyltransferase
A0A0Z8HVB3	1.395864	OsmC-like protein
A4VXE3	1.395286	Transcriptional regulator/sugar kinase
A0A0Z8CXG9	1.395255	Geranylgeranyl pyrophosphate synthase
A0A0Z8N6Z5	1.39416	Enoyl-CoA hydratase
A0A2I5KNB7	1.39313	DUF1727 domain-containing protein
A4VZG5	1.392693	Glutamyl-tRNA(Gln) amidotransferase subunit A
A0A0Z8WGY7	1.391698	Asparagine--tRNA ligase
A0A0Z8FH86	1.389526	Mevalonate kinase
A0A0Z8CD01	1.389467	Sugar ABC transporter periplasmic protein
A0A0Z8JEF3	1.386678	Membrane protein
D5AGZ3	1.386093	Tellurite resistance protein,TelA
A0A0N0VC74	1.385079	Transcriptional regulator
A0A4T2GR37	1.384723	Homoserine kinase
U5UCB3	1.383204	Nitrate/sulfonate/bicarbonate ABC transporter periplasmic protein
A4W235	1.382592	Shikimate dehydrogenase (NADP(+))
A0A140EXH1	1.382574	Glutamine ABC transporter substrate-binding protein
A0A0Z8RTZ3	1.381537	Uncharacterized phage-encoded protein
A0A2I5KS69	1.378668	ABC transporter ATP-binding protein
A0A0Z8D1F7	1.378174	ABC-2 family transporter protein
A4W125	1.378045	Methylenetetrahydrofolate--tRNA-(uracil-5-)-methyltransferase TrmFO
A0A426TEW5	1.377221	UPF0176 protein EI998_05090
A0A2K1T1D0	1.377207	MTS domain-containing protein
G7SJC2	1.376945	-
A0A0M9FHX7	1.376264	XRE family transcriptional regulator
A0A0Z8M2M6	1.375591	Glutamine amidotransferase
A0A0Z8FWL7	1.374438	Ribonuclease Z

A0A2K1T2G1	1.373009	Chemotaxis protein
A0A4T2GII4	1.372816	ATP synthase subunit beta
A0A0Z8EMZ0	1.371372	LysR family transcriptional regulator
A1YSF3	1.37074	Aminotransferase, class I and II
A0A075SNC6	1.369707	Ribose-phosphate pyrophosphokinase
A4VTQ9	1.368997	Uncharacterized protein
A0A075SGD7	1.368285	Uncharacterized protein
A0A4T2GJ40	1.366871	Acylphosphatase
G7SNU9	1.366175	-
A4VT89	1.366136	Galactose-1-phosphate uridylyltransferase
A0A0Z8GSD4	1.366109	Copper-exporting ATPase
A0A2K1SYM6	1.365741	ATPase AAA
A0A426TF03	1.363563	Threonine synthase
D5AGQ6	1.361067	Tyrosine-protein phosphatase
A0A0Z8G0E8	1.360567	Prophage maintenance system killer protein
A0A0Z8FF88	1.360118	Methionine import ATP-binding protein MetN
A0A426TD10	1.360097	MBL fold metallo-hydrolase
A0A4P7WQ87	1.35998	Penicillin-binding protein
A4VTS0	1.359838	ATP-dependent 6-phosphofructokinase
A0A1X9I1X3	1.35825	Uncharacterized protein
A0A2I5N4I6	1.35677	Site-specific integrase
A4VUP9	1.355744	Hypothetical cytosolic protein
A0A0Z8TY67	1.355714	Pyruvate/2-oxoglutarate dehydrogenase complex, dehydrogenase (E1) component, alpha subunit
A0A116LUY2	1.355099	DnaD and phage-associated domain protein
A0A0Z8IY35	1.353598	Major tail protein
A0A075SDC1	1.353391	Spermidine/putrescine ABC transporter substrate-binding protein
A0A123SN79	1.35289	Spermidine/putrescine-binding periplasmic protein
A4VWZ2	1.352333	Probable manganese-dependent inorganic pyrophosphatase
A0A4T2GZI7	1.351629	Lipoprotein
A0A123W293	1.351516	Ribonuclease M5
A0A0N0DPU2	1.35086	Nicotinate phosphoribosyltransferase
A0A1P8VS09	1.350642	Chain length determinant protein Wzd
A0A116M841	1.350437	Membrane carboxypeptidase
D5AH31	1.349566	Uncharacterized protein
A0A0M9FFB0	1.346085	Segregation and condensation protein A
A0A0Z8BJL5	1.345198	TM2 domain-containing protein
A0A0Z8CTJ9	1.344718	Cell wall biosynthesis glycosyltransferase
A4VSE5	1.343664	Putative competence-damage inducible protein
D5AF98	1.342903	Putative transcriptional regulator
A0A075SM43	1.339971	Membrane protein
A4VV15	1.339948	Predicted Rossmann fold nucleotide-binding protein involved in DNA uptake

A0A2I5N153	1.338864	Adenylosuccinate lyase
D5AJI0	1.336994	Putative diaminopimelate decarboxylase
A0A2I5KD53	1.336829	Uncharacterized protein
D5AGG0	1.33596	Glutathione reductase
A0A0Z8D2Q4	1.335657	Transcriptional regulator
A0A0M9FET3	1.335289	DhaKLM operon coactivator DhaQ
A0A0M9FNT9	1.334655	ABC transporter
D5AGQ4	1.334422	Cps2B
A0A116KB70	1.334014	UDP-N-acetylglucosamine 1-carboxyvinyltransferase
A0A0Z8DS84	1.333954	Histone acetyltransferase HPA2-like acetyltransferase
D5AHM8	1.333824	Dihydroorotase
A0A2K1T0H1	1.332572	UDP-galactose-4-epimerase
A0A0Z8IWS5	1.330656	Probable tRNA sulfurtransferase
A0A0N0VCC1	1.330561	GntR family transcriptional regulator
A0A0K2E8J9	1.330476	Uncharacterized protein
D5AFI0	1.330221	Putative folylpolyglutamate synthetase
A0A0Z8FJ25	1.32968	Ribosomal RNA small subunit methyltransferase G
A0A0Z8K9L2	1.329122	Gp42
A0A0N0DQR3	1.328555	Type I restriction enzyme R Protein
A4VX69	1.328008	Cell division protein FtsI/penicillin-binding protein 2
A0A0Z8EE19	1.326254	Membrane protein
A0A0Z8CTM5	1.326055	LHH domain-containing protein
A0A2K1SK08	1.325716	tRNA pseudouridine synthase B
A0A4V4RWG5	1.325477	F5/8 type C domain-containing protein
A0A0Z8DEE3	1.325237	Putative gluconeogenesis factor
A0A2K1T0X2	1.323552	Glycosyl transferase
A0A0Z8MHH3	1.323081	Cell division protein DivIB
A0A116N7S9	1.322176	3-isopropylmalate dehydrogenase
A0A0Z8EWE9	1.321888	ABC transporter permease
A0A4P7WNV8	1.321518	Homoserine dehydrogenase
A0A0K2E8K2	1.321331	DNA-binding protein
D5AGX3	1.320745	D-alanine--D-alanyl carrier protein ligase
G7SET1	1.319288	Putative amino acid ABC transporter, ATP-binding protein
A0A0Z8NS16	1.318101	3-ketoacyl-ACP reductase
A0A0Z8RQ12	1.317235	HAD superfamily hydrolase
A0A0Z8X6E1	1.313996	Mrr_cat domain-containing protein
A0A0Z8BXC4	1.313931	HAD superfamily hydrolase
A0A2I5KH23	1.312646	Histidine phosphatase family protein
A0A2I5N2J2	1.311865	DUF2974 domain-containing protein
A0A0Z8KNS0	1.311205	Glutathione S-transferase
A0A0N0VAX5	1.309715	DeoR faimly transcriptional regulator
A0A4T2H0W7	1.309404	Oligoendopeptidase F
D5AFX4	1.3083	Heat-inducible transcription repressor HrcA
A0A0Z8FG45	1.308289	Competence associated protein

A0A0Z8J387	1.307653	Ribonuclease HII
A0A0Z8ER14	1.307184	Transglutaminase-like protein
A0A0Z8LUX3	1.306772	HAD superfamily hydrolase
A0A2K1T1U6	1.306619	Protease
A4VTK4	1.305177	UDP-N-acetylglucosamine--N-acetylmuramyl-(pentapeptide) pyrophosphoryl-undecaprenol N-acetylglucosamine transferase
A4VTT5	1.305107	Gamma-glutamyl phosphate reductase
A0A2K1SXK8	1.305036	Dihydrofolate synthase
D5AHA4	1.304801	Aminotransferase, class V
D5AIG9	1.303973	Uridylate kinase
A0A0M9FGI5	1.302903	Histidine kinase
A0A0M9FIS3	1.301664	GTP-sensing transcriptional pleiotropic repressor CodY
A0A0Z7Z8M0	1.301576	Pyruvate formate-lyase-activating enzyme
A0A0Z8G5N0	1.300245	SAM-dependent methyltransferase
A0A123T041	1.299308	ADP-glucose pyrophosphorylase
A0A116NA42	1.299168	Xaa-Pro dipeptidase
A4VZI7	1.29847	tRNA(Met) cytidine acetate ligase
A0A0Z8H1P8	1.297986	Multifunctional fusion protein
A0A0Z8D1B8	1.297512	Uncharacterized protein
A0A0Z8E3V9	1.296244	UTP--glucose-1-phosphate uridylyltransferase
A0A0M9FN71	1.296175	GntR family transcriptional regulator
A0A0Z8F9T8	1.293982	UPF0210 protein CWI26_01740
A0A0M9FP27	1.293943	Ribosomal RNA small subunit methyltransferase H
A0A0Z8IEU8	1.29393	Polysaccharide biosynthesis protein/ rhamnosyl transferase
A0A0Z8D8X4	1.293351	Membrane protein
D5AFX9	1.29256	Amidase
A0A2K1SWX5	1.291033	Tryptophan--tRNA ligase
D5AJ78	1.290503	HPr kinase/phosphorylase
A0A426TD39	1.290462	Phosphopantothenate--cysteine ligase
G7SFT1	1.289497	SAM-dependent methyltransferase
A0A0Z8HM93	1.288811	UDP-N-acetylglucosamine--peptide N-acetylglucosaminyltransferase stabilizing protein GtfB
A0A0Z8LFW1	1.288046	DegV family protein
U5UFM8	1.287768	Uncharacterized protein
A0A0Z8XIC8	1.287756	Queuine tRNA-ribosyltransferase
A4VXD9	1.286775	Alanine racemase
A0A3R8NMD3	1.286504	Phosphoglucosamine mutase
A0A2K1SK19	1.283654	Riboflavin biosynthesis protein
D5AK28	1.28271	Cysteine--tRNA ligase
A0A0Z8KAI0	1.280534	Ribosome biogenesis GTPase A
A0A4T2HDG5	1.279703	GMP synthase [glutamine-hydrolyzing]
A0A426THS7	1.278981	Peptidase T
A0A116KWZ8	1.278483	Transcriptional regulator
A0A0H3N6K3	1.278457	RmuC family protein

A0A4V0EBB3	1.276894	Uncharacterized protein
A0A4T2GU76	1.276544	Aspartate carbamoyltransferase
A0A0M9FGK7	1.274913	Glutamate racemase
A0A116KLL4	1.273968	ABC transporter ATP-binding protein
A4VTM4	1.273535	Bifunctional protein FolD
A4VUR8	1.271089	ABC-type antimicrobial peptide transport system, ATPase component
A0A0M9FEC1	1.270364	RNA-binding protein
A0A2K1T1N3	1.269305	Regulatory protein RecX
A4VUY3	1.269161	Methyltransf_11 domain-containing protein
D5AFQ6	1.268757	Signal peptidase I
A0A2K1T123	1.268377	ArsR family transcriptional regulator
Q9S5D7	1.267667	Mob
A0A0Z8E1R4	1.267049	ABC transporter ATP-binding protein
A0A2K1T0W1	1.266466	Tagatose 1,6-diphosphate aldolase
D5AJM3	1.265458	Polyprenyl synthetase
A0A0Z8ULY2	1.265009	2,3-bisphosphoglycerate-dependent phosphoglycerate mutase
A0A123SXF2	1.263586	Ribosome-binding ATPase YchF
A0A3R8R773	1.260657	YSIRK-type signal peptide-containing protein
A0A4T2GKR5	1.260128	Chromosomal replication initiator protein DnaA
A0A0Z8D9T4	1.258996	DNA polymerase III subunit delta
A0A2K1T0W0	1.256873	Histidine triad protein
A0A0Z8KAC5	1.256113	Gp37
A0A0Z8CMI1	1.255649	Ser/Thr protein phosphatase family protein
A0A0Z8CT14	1.255457	Membrane-associated phospholipid phosphatase
A0A0Z8I5X3	1.254042	Adenosine deaminase
A0A0Z9K0W7	1.253362	Pyruvate dehydrogenase
A0A0Z8CI31	1.253356	LemA family protein
A0A2K1T2R5	1.252995	ABC transporter substrate-binding protein
A0A123SPI7	1.252172	Uridine phosphorylase
A0A2K1T2N7	1.251925	Threonine synthase
A0A0Z8FC74	1.251069	Peptide deformylase
D5AFW6	1.249117	Diphosphomevalonate decarboxylase
A0A0Z8CTW4	1.248674	PTS glucose/maltose transporter subunit IIBCA
D5AIU7	1.247425	Uncharacterized protein
A0A0M9FMJ3	1.246911	Galactokinase
A0A0Z8FMN6	1.245994	Multidrug ABC transporter ATPase
A0A3R8REL0	1.244376	tRNA pseudouridine synthase B
A4VVW6	1.242671	Glycosyltransferase
A0A0M9FGX7	1.241709	DNA-binding protein
A0A0Z8EYH1	1.241442	Beta-lactamase class A
A0A2K1SYG5	1.240281	Homoserine dehydrogenase
D5AHS9	1.237205	RelA/SpoT
A0A2K1SX52	1.236331	Glycosyl transferase

A0A116MCI4	1.23447	Response regulator
A0A2I5KH71	1.233691	NH(3)-dependent NAD(+) synthetase
A0A116R0U2	1.232723	Malonyl CoA-acyl carrier protein transacylase
A4VUW3	1.230477	Signal recognition particle GTPase
A0A0Z8MBF3	1.229203	Phosphosugar isomerase
D5AIW0	1.228784	ABC transporter, ATP-binding protein
D5AIE7	1.228526	Predicted SAM-dependent methyltransferase
A0A0Z8EP50	1.228158	Elongation factor Ts
A0A160JQD3	1.22745	Glucokinase
D5AFW8	1.227417	Isopentenyl-diphosphate delta-isomerase
D5AFP1	1.227059	Tagatose-6-phosphate kinase
A0A4T2H0G2	1.226588	Peptide deformylase
A0A1P8VRN5	1.226189	Tyrosine-protein kinase Wze
A0A0Z8II17	1.225679	4-alpha-glucanotransferase
A0A0Z8EST8	1.225023	Aminotransferase
A0A3R8SA74	1.221356	GTPase Era
A0A0H3MX61	1.219301	L-threonine dehydratase
A4VWL9	1.218783	Epoxyqueuosine reductase QueH
A0A0Z8AP84	1.218276	Transcriptional regulator/sugar kinase
A0A0Z8MPG5	1.217413	30S ribosomal protein S7
A0A0Z8H391	1.217288	ADP-ribose pyrophosphatase
A0A4T2H2D6	1.215655	Acetate kinase
D5AG85	1.213878	Cof protein: HAD-superfamily hydrolase, subfamily IIB
G7SK07	1.213841	-
A0A0N0VBX6	1.213332	HD superfamily phosphohydrolase
A0A0M9FH17	1.213138	Ribonuclease P protein component
A0A116NBA1	1.209215	UPF0176 protein EJA00_11420
A0A426TJD6	1.20729	HPr kinase/phosphorylase
A0A0Z8TLS8	1.205006	Uncharacterized protein
A0A0Z8LUH7	1.204494	Small ribosomal subunit biogenesis GTPase RsgA
A0A0Z8AQE9	1.204462	Excalibur domain-containing protein
D5AH72	1.203816	Cobalt ABC transporter ATP-binding protein
A0A426TKC6	1.203657	ATP-binding protein
A0A2I5KCH0	1.203411	DNA repair protein RecO
D5AJJ1	1.202556	Uncharacterized protein
A0A123U4W4	0.832942	50S ribosomal protein L28
A0A3R8TEB9	0.831137	Ketosteroid isomerase
A0A075SU63	0.830563	Maltodextrose utilization protein MalA
A0A0Z8HVW5	0.826511	Zn-finger
A0A116QYF5	0.826478	Multidrug ABC transporter ATP-binding protein
A0A0Z8V873	0.825315	Glycerol uptake facilitator protein, putative
D5AHS5	0.822244	ABC transporter related protein
A0A0Z8XTP6	0.821262	KAP NTPase domain-containing protein
A0A3R8SBL2	0.819909	GAF domain-containing protein

A4VYQ2	0.818837	30S ribosomal protein S17
A0A0Z8JQD8	0.817478	Endonuclease MutS2
A0A0M9FJW0	0.816741	ABC transporter substrate-binding protein
A0A426TE53	0.816146	DUF956 family protein
D5AJA8	0.815047	Polysaccharide deacetylase
A0A0Z8G0G7	0.813803	Uncharacterized protein
A0A0Z8JQ04	0.811905	Protein DltB
A0A0Z8ILD2	0.811422	Ribonucleases G and E
A0A0Z8J9G3	0.811046	Integrase
A0A4P7WQM9	0.810579	MFS transporter
A0A4T2GL14	0.80817	Holo-[acyl-carrier-protein] synthase
D5AF13	0.807025	Beta-glucosidase A
A0A0Z8ML39	0.806958	Sugar ABC transporter periplasmic protein
A0A4T2GM07	0.806447	NAD(P)/FAD-dependent oxidoreductase
A0A123S845	0.806371	Peptidyl-tRNA hydrolase
A0A0Z8IIF6	0.804764	ABC transporter permease
A0A426TID8	0.803734	ABC transporter ATP-binding protein
A0A2K1T257	0.803557	Clp protease ClpX
A0A0Z8H6W5	0.803477	Acetolactate synthase 3 regulatory subunit
A0A426TDN5	0.803244	Transcription termination/antitermination protein NusA
G7SDM1	0.802239	Cell division initiation protein
A0A0Z8FK93	0.80167	Phage Holliday junction resolvase
A0A116RJ74	0.79882	MutT/NUDIX hydrolase family protein
A0A116S2Y0	0.796238	50S ribosomal protein L24
A0A426G7H7	0.792878	POLAc domain-containing protein
A0A0Z8ET79	0.792249	tRNA-dihydrouridine synthase
I6MEY8	0.790545	Muramidase-released protein
A0A0Z8J022	0.790234	Amino acid ABC transporter ATP-binding protein
A0A0Z8CX33	0.789906	Adenylate kinase
A0A0Z8EQP7	0.788755	Ribosome-associated heat shock protein implicated in the recycling of the 50S subunit
A0A0Z8TV46	0.787294	4-oxalocrotonate tautomerase
A0A0M9FFA2	0.785891	30S ribosomal protein S1
D5AEQ5	0.78533	Response regulator
A0A0Z8I025	0.785226	Prolyl-tRNA synthetase
A0A116NPV2	0.78169	ATP-dependent helicase/nuclease subunit A
A0A123UB04	0.778594	LPXTG-motif cell wall anchor domain-containing protein
A0A4T2GPG1	0.777958	ATP-cone domain-containing protein
A0A116NK51	0.774078	Signal peptidase I
A0A2S2F6J5	0.773861	5-methyltetrahydropteroyltriglutamate--homocysteine methyltransferase
A0A1P8VRB2	0.773013	Glycosyltransferase
A0A0Z8D0B1	0.770979	5-formyltetrahydrofolate cyclo-ligase
A0A0Z8HKR7	0.770665	Minor tail protein Gp26

A0A0Z8G053	0.770295	Membrane protein
A4VT67	0.768385	Fructose-bisphosphate aldolase
A0A0Z8KF64	0.767182	ATP-binding membrane protein
A0A4T2GTY4	0.766097	LacI family transcriptional regulator
A0A2I5KEM8	0.763805	Uncharacterized protein
A0A426TGV8	0.763282	Energy-coupling factor transporter ATP-binding protein EcfA
A0A116MPE9	0.761939	ParB/RepB/Spo0J family partition protein
A0A0Z8E784	0.761174	Anaerobic ribonucleoside-triphosphate reductase-activating protein
A0A140EXL5	0.759421	Protein rep
A0A0Z8SIY5	0.757633	Multidrug ABC transporter ATP-binding protein
D5AJ62	0.757385	Transcriptional regulator, PadR family protein
A0A4T2H4W7	0.757006	Glutamate--tRNA ligase
A0A0Z8JRG2	0.756832	Ribosome-binding factor A
A0A123SMW7	0.755769	ABC-type enterochelin transport system, ATPase component
A0A2K1SJI8	0.755219	PadR family transcriptional regulator
A0A0Z8P396	0.75475	Cell wall surface anchor family protein
A0A0Z8GDW2	0.754628	Polar amino acid ABC transporter ATPase
A0A0Z8QH43	0.754043	Subtilisin-like serine protease
A0A0M9FJR7	0.752199	Amidase
A0A0Z8IVB5	0.750201	Transcriptional regulator
A0A0Z8F7W1	0.749576	Acyl-coenzyme A synthetases/AMP-(Fatty) acid ligase
A0A345S4Q3	0.748438	Copper ABC transporter permease
D5AI87	0.747805	Uncharacterized protein
A0A2I5KN05	0.747138	DUF2188 domain-containing protein
A4VUD0	0.746514	MRP
D5AIP3	0.746313	Ferrous iron transport protein A
D5AJW6	0.741308	GCN5-related N-acetyltransferase
A4VT80	0.739635	50S ribosomal protein L19
A0A426T2D4	0.739575	Uncharacterized protein
A0A0Z8E384	0.738044	Phosphotransferase system, mannose/fructose/N-acetylgalactosamine-specific component IIC
A0A2I5KLQ9	0.737622	Replication protein RepB
A0A1X9I1N1	0.736767	Abortive infection protein AbiGI
A0A4T2GJP3	0.735257	SP_1767 family glycosyltransferase
A0A0Z8T129	0.735245	Ribokinase family sugar kinase
A0A0M9FER6	0.734951	ArsR family transcriptional regulator
A0A0H3MXD2	0.734021	Putative preprotein translocase subunit
G7SJH9	0.732566	-
A0A123U470	0.732275	Predicted transcriptional regulator with C-terminal CBS domains
A0A345S3L9	0.731942	Permease IIC component
A0A0Z8GWR9	0.730304	Membrane protein
G7SHU0	0.728589	Membrane protein

A0A116L8P1	0.727968	DUF3885 domain-containing protein
A0A4T2GP62	0.727779	Aminotransferase DegT
A0A0Z8CPL4	0.72739	Muramidase-released protein
A0A0Z8W959	0.727112	Cytosine-specific methyltransferase
D5AHB7	0.726704	Carbamoyl-phosphate synthase small chain
A0A0Z8DYH8	0.726547	Domain of uncharacterized function (DUF3173)
A0A3R8LWE4	0.726106	ImmA/IrrE family metallo-endopeptidase
A0A2S2F687	0.725575	DUF1304 domain-containing protein
A0A4T2GQZ0	0.725002	Dihydroorotate dehydrogenase
A0A116QZQ1	0.723999	Membrane protein
A0A0Z8W6X8	0.723806	Uncharacterized protein
A0A116L4S4	0.720536	Cytosine-specific methyltransferase
A0A0Z8FXE0	0.720514	Uncharacterized protein
A0A2K1SXY6	0.720258	Toxin YoeB
A0A4T2GR34	0.719406	CYTH domain-containing protein
A0A0Z8UFJ3	0.719397	Copper-transporting ATPase
D5AFZ9	0.718543	Regulatory protein, TetR
A0A0Z8KKK0	0.717925	Uncharacterized protein
A0A0Z8W6V7	0.716382	PTS lactose transporter subunit IIB
N1JUU3	0.715872	Antioxidant protein
A0A2I5KDM0	0.715831	Guanylate kinase
A0A4T2GLY4	0.714555	Phosphatidate cytidylyltransferase
A0A3R8SDL1	0.71423	Terminase large subunit
D5AJ91	0.71338	GCN5-related N-acetyltransferase
A0A0Z8HCC8	0.713204	GNAT family N-acetyltransferase
A0A123STR2	0.712566	RNA polymerase sigma factor, sigma-70 family
A0A426TID2	0.712049	ROK family glucokinase
A0A116RN02	0.711843	Uncharacterized protein
A0A126ULT1	0.711656	Amino acid ABC transporter permease
A0A0Z8B0D0	0.710137	Collagen-binding protein A
A0A2I5KP67	0.70999	UDP-N-acetylenolpyruvoylglucosamine reductase
A0A0F6UY66	0.709168	Uncharacterized protein
A0A0Z8H697	0.708143	Transcriptional regulator
A0A0Z8DVY6	0.706967	Response regulator
A0A0Z8JC04	0.706521	Putative DNA replication protein
A0A0Z8BEB1	0.706144	4-oxalocrotonate tautomerase
A0A345S3G3	0.706142	NCS2 family permease
A0A0M9FG87	0.705072	Acetolactate synthase
A0A0M9FJJ3	0.701278	Ketosteroid isomerase
A0A116RIH3	0.701021	Agglutinin receptor
A0A0Z8C555	0.700845	Thiol-disulfide isomerase and thioredoxin
A0A2I5KP48	0.700776	Replication initiator protein
A0A426TNY8	0.699482	LPXTG cell wall anchor domain-containing protein
A0A0Z8QEK5	0.699173	[Ribosomal protein S18]-alanine N-acetyltransferase

A0A4T2GRJ7	0.698793	DNA starvation/stationary phase protection protein
A0A0Z8XPG5	0.697378	Integrase
A0A123YMD2	0.697052	BlpT protein, fusion
A0A116L321	0.696694	Type I restriction-modification system methyltransferase subunit
A0A0Z8UJ91	0.696306	PIN_8 domain-containing protein
A0A116N2V8	0.696012	Uncharacterized protein
U5UDR7	0.695356	Uncharacterized protein
A0A2I5KS00	0.694951	Cell division protein FtsK
A0A0Z8FMU5	0.69376	DEAD-box ATP-dependent RNA helicase CshA
A0A116NG44	0.69317	Precorrin-6x reductase
A0A0H3N1W3	0.693092	50S ribosomal protein L33
A0A4T2H292	0.692691	Lipoprotein
A0A3R8T158	0.692015	N-acetyltransferase
A0A4V4RW78	0.691135	Uncharacterized protein
A0A0Z8FLC6	0.690806	Uncharacterized protein
A0A2I5KMP1	0.690136	Sugar ABC transporter permease
A4VT05	0.689831	50S ribosomal protein L33
A0A123TM48	0.685703	Initiation-control protein YabA
A0A2K1SX31	0.685636	Ribonucleoside-triphosphate reductase
A0A160JP73	0.685445	Histidine kinase domain-containing protein
G7SL12	0.68324	-
A0A426GA42	0.681932	ABC transporter ATP-binding protein
A0A0Z8F659	0.68117	Transcriptional regulator
G7SMV6	0.681006	-
A0A0Z8FJS3	0.680965	Aminoglycoside-2"-adenylyltransferase
A4VXH2	0.68057	Ribosome-binding factor A
A0A0Z8F8E7	0.678885	Phosphotransferase system cellobiose-specific component IIA
A0A0M9FK22	0.677615	Uncharacterized protein
D5AFR7	0.676394	Acetyltransferase, GNAT family protein
A0A3R8R6S0	0.675091	YSIRK-type signal peptide-containing protein
A0A4T2GLE2	0.674357	Glyceraldehyde-3-phosphate dehydrogenase
A0A116L141	0.671903	Phage protein
A0A2I5KIV0	0.671529	Type II toxin-antitoxin system HicA family toxin
A0A123U9P8	0.671394	Uncharacterized protein
A0A116MA07	0.66997	Branched-chain amino acid transport system carrier protein
A0A123U809	0.668174	Uncharacterized protein
A0A0Z8HZ35	0.666125	Replication protein
A0A0Z8H622	0.665762	Uncharacterized protein
A0A3R8T814	0.665324	Glutamine--fructose-6-phosphate aminotransferase [isomerizing]
A0A2I5N1B6	0.665104	50S ribosomal protein L17
A0A116PZE2	0.663727	LPXTG cell wall surface protein
A0A0Z8FSN4	0.663037	ABC transporter permease

A0A0Z8M0U7	0.662736	Superfamily II DNA/RNA helicase
A0A0Z8JFL4	0.661623	Phenylalanine--tRNA ligase beta subunit
A0A160JNU5	0.660631	Cell surface protein
A0A4T2GIZ1	0.660505	Oligopeptide ABC transporter substrate-binding protein
A0A0Z8IDB4	0.660064	tRNA-dependent lipid II-Ala--L-alanine ligase
A0A116MA70	0.660028	PhnA protein
A0A0Z8R278	0.659795	Glycosyl hydrolase
D5AFP0	0.659741	NAD(P)-binding Rossmann-like domain protein
A0A0Z8N4T0	0.6586	Lantibiotic efflux protein
A0A4T2HAY6	0.657141	CCA-adding enzyme
A4VT01	0.657095	Translation initiation factor 2 (IF-2 GTPase)
M1VJZ4	0.65701	Uncharacterized protein
A0A2K1T2P9	0.656824	Membrane protein
A0A0Z8EZ46	0.656113	Hypothetical phage protein, putative
A0A426TE06	0.654269	Translation initiation factor IF-2
A0A0Z8E6I0	0.654115	Lead, cadmium, zinc and mercury transporting ATPase
A0A0Z8T0Y0	0.653687	Phosphatase
A0A0Z8HT53	0.653438	Glyoxalase
A0A0Z8EMW9	0.652152	Cytosine-specific methyltransferase
A0A1C9IF04	0.651369	Uncharacterized protein
G7SLJ8	0.651344	-
A4VVX2	0.65048	Uncharacterized protein
A0A075SJ48	0.650262	Uncharacterized protein
A0A4T2H057	0.649131	SMI1/KNR4 family protein
A0A4T2GR62	0.64913	50S ribosomal protein L10
A0A0Z8LW04	0.648297	Subtilisin-like serine protease
A0A426THS4	0.64814	Iron export ABC transporter permease subunit FetB
M1VRI6	0.646443	NAD dependent epimerase/dehydratase family protein
A0A0M9FFG6	0.645578	Thioesterase
A4VVB6	0.645504	30S ribosomal protein S20
A0A2I5N1K7	0.645459	Uncharacterized protein
A0A3R8SKU4	0.644654	Mid-cell-anchored protein Z
A0A4T2H448	0.64394	Elongation factor Ts
A0A0Z8HYA1	0.643404	Uncharacterized protein
F6L052	0.643291	Ribosomal protein L7/L12
A0A0Z8MRH2	0.642359	Uncharacterized protein conserved in bacteria
A0A0M9FMX2	0.641546	Amino acid ABC transporter permease
A0A426TFZ5	0.641413	DUF438 domain-containing protein
A0A4T2GV79	0.639712	DUF2813 domain-containing protein
A0A0Z8JIE0	0.639464	Uncharacterized conserved protein
A0A0Z8G0K9	0.638664	Lpxtg-motif cell wall anchor domain-containing protein
A0A0F6S2I8	0.638579	Choline kinase
A0A3R8SWW8	0.637608	VOC family protein
A0A0Z8P7P9	0.637595	Accessory Sec system protein Asp3

A0A4T2GNZ6	0.637416	Ribosome hibernation promoting factor
A0A0Z8I8T1	0.636589	Predicted ATP-binding protein involved in virulence
A0A123T3K9	0.636259	Uncharacterized protein
A0A3R8SBJ0	0.634747	Thymidylate kinase
A0A0H3MXF6	0.633985	Surface-anchored DNA nuclease
A0A0N0DLY8	0.62849	Alpha-1,4 glucan phosphorylase
A0A0Z8JJX3	0.628325	Uncharacterized protein
A0A0Z8LHA6	0.624998	5-methyltetrahydropteroyltriglutamate--homocysteine methyltransferase
A0A0Z8HE15	0.621498	Plasmid replication protein Rep and AAA-class ATPase domain protein
A0A0Z8L247	0.621324	Glutamine ABC transporter substrate-binding protein
A0A140EXG3	0.620862	Metallophosphatase
A0A0M9FJX4	0.61946	Uncharacterized protein
A0A0M9FE30	0.619422	Addiction module toxin, HicA family
A0A0Z8LZ07	0.616869	Prophage LambdaSa2, lysin
A0A075SKX2	0.616754	2', 3'-cyclic nucleotide 2'-phosphodiesterase
A0A116LX99	0.615342	Cof family hydrolase
B5UAE3	0.61459	Putative pilus subunit protein
D5AHR5	0.614049	Galactose-6-phosphate isomerase subunit LacA
A0A0Z8GNJ7	0.61396	Dehydrogenase
D5AI73	0.613245	Oxidoreductase
A0A0Z8CJT2	0.612746	Transcriptional regulator
A0A426TEQ0	0.612516	DNA repair protein RecN
A4VYQ6	0.611686	30S ribosomal protein S8
D5AF82	0.610918	N5-carboxyaminoimidazole ribonucleotide mutase
A0A123V8I4	0.610901	HAD-superfamily hydrolase
A4VSG4	0.608923	50S ribosomal protein L14
A0A4V4RY00	0.608136	Winged helix-turn-helix transcriptional regulator
A0A0Z8JRL6	0.607443	Lipopolysaccharide glycosyltransferase
A0A4V0EWY0	0.607234	Prophage ps3 protein 13
A0A2K1SXZ9	0.605929	Glyoxalase
G7SGP0	0.60575	Surface antigen SP1
A0A345S4A0	0.605321	VOC family protein
A0A3R8R8V6	0.603806	ABC transporter ATP-binding protein
A0A2S2F7D9	0.60349	MarR family transcriptional regulator
A0A346FW03	0.602869	Integral membrane protein
A0A116LYL1	0.602332	Chloride channel protein EriC
A0A116L531	0.601396	Replication protein RepR
A0A0M9FML2	0.600267	Ribosome hibernation promoting factor
A0A0N1J3Y7	0.599976	TIR domain-containing protein
A0A0Z8CHJ2	0.599386	Response regulator
A0A4V0ETH3	0.598775	Uncharacterized protein
A0A4V6U7B1	0.598056	MerR family transcriptional regulator

A0A0Z8EES2	0.598011	Isocitrate dehydrogenase [NADP]
A0A0Z8NP66	0.597777	O-antigen and teichoic acid export protein
A0A4V0ECV3	0.597357	Phosphomethylpyrimidine kinase type-1
B0M0G9	0.595638	Uncharacterized protein
A0A0Z8FBX5	0.595448	Elongation factor P
A0A4T2GUU2	0.594159	Transporter substrate-binding domain-containing protein
A0A116NFH5	0.593787	Glutamate-1-semialdehyde 2,1-aminomutase
A0A0K2E3D6	0.593518	50S ribosomal protein L36
A0A4T2GWQ5	0.591321	Translation initiation factor IF-2
A0A4V0E4T2	0.590418	UPF0223 protein Spy49
A0A116QMN7	0.587902	Tagatose-6-phosphate kinase
A0A123U767	0.587317	Metal-sulfur cluster biosynthetic protein
A0A3R8M1M3	0.587035	LPXTG cell wall anchor domain-containing protein
A0A3S6JTK7	0.586581	DUF3666 domain-containing protein
A4VSS5	0.586115	Uncharacterized protein conserved in bacteria
A0A4T2H489	0.583995	ABC transporter permease
A0A0Z8J8I4	0.58235	Galactose-6-phosphate isomerase subunit LacB
A0A0Z8DCW0	0.577933	Site-specific recombinase, phage integrase family
A0A0M9FF10	0.577259	Uncharacterized protein
A0A123UBW1	0.574236	30S ribosomal protein S13
A0A4T2GQB3	0.573109	Single-stranded-DNA-specific exonuclease RecJ
A0A0Z8MBY0	0.572449	Type I restriction enzyme R Protein
A4W3Q2	0.571166	30S ribosomal protein S6
A0A426TD71	0.569764	30S ribosomal protein S9
A0A123TLT1	0.568987	Flavodoxin
A0A123VBJ7	0.567104	Glutamine ABC transporter substrate-binding protein
A0A0Z8FT18	0.565207	Twin-arginine translocation pathway signal
U5KKI1	0.564984	Cps19K
A0A4V0EV03	0.564379	PTS system IIABC component
A0A4P7WNY7	0.564217	YSIRK-type signal peptide-containing protein
A0A426G4B6	0.562187	DNA mismatch repair protein MutH
A4VXW0	0.558306	Transcription termination/antitermination protein NusG
A0A0Z8P7D6	0.555902	Protein translocase subunit SecA
A0A0Z8GFG1	0.555602	Uncharacterized protein
A0A0N0VC94	0.554845	PTS sucrose transporter subunit IIABC
A0A140EXG9	0.553347	Keto-deoxy-phosphogluconate aldolase
A0A0Z8JRR2	0.550675	Putative ATP-dependent endonuclease of the OLDfamily
A0A2K1SY56	0.549575	Amino acid ABC transporter permease
A0A0Z8LGJ0	0.547086	Copper-transporting ATPase
A0A0Z8B2A5	0.546327	5'-nucleotidase
A0A116K597	0.541784	Phosphotransferase system, mannose/fructose/N-acetylgalactosamine-specific component IIB
S5PZS7	0.541371	Site-specific DNA-methyltransferase (adenine-specific)
A0A0Z8BY06	0.540874	Amino acid ABC transporter periplasmic protein

D5AFF1	0.540407	Uncharacterized protein
A0A0M9FDK6	0.538855	Transposase
D5AGC3	0.536051	Surface protein from Gram-positive cocci
A0A0N1J3P0	0.535749	DUF1801 domain-containing protein
A0A4T2H6Y4	0.535435	Pneumococcal-type histidine triad protein
A0A126UNE1	0.535336	Transposase
A0A0Z8LE80	0.53489	Surface-anchored protein
D5AFJ8	0.533912	Transcriptional regulator, glutamine synthetase repressor
A0A4T2H0Y5	0.533259	YSIRK-type signal peptide-containing protein
S6B433	0.525226	RecN protein
A0A0Z8MCC7	0.525032	Peptidoglycan-binding LysM
A0A123TR22	0.52376	C protein alpha-antigen
A0A4T2GJP6	0.517526	50S ribosomal protein L11
A0A4T2GNA0	0.515115	Regulatory protein RecX
A0A3Q8B7S0	0.515057	Cell surface protein
A0A2Z4PJY8	0.513007	Citrate synthase
A0A0M9FEK7	0.511104	Cold-shock protein
A0A116NAA3	0.508534	Methyl-accepting chemotaxis protein
A0A4P7WMK7	0.506213	Exodeoxyribonuclease 7 large subunit
M1VNS6	0.505917	DNA repair ATPase
A0A426TDA1	0.500657	Amino acid ABC transporter ATP-binding protein
A0A0Z8E2P3	0.500305	Type IV secretory pathway, VirD4 component
A0A116MNT4	0.497993	Uncharacterized protein
A0A0Z8GPZ6	0.496026	Cytosine-specific methyltransferase
G7SEJ0	0.495431	Uncharacterized protein
A0A2I5N1S5	0.493914	Beta-carotene 15,15'-monooxygenase
A0A426TII7	0.490629	Aconitate hydratase
A0A2I5N579	0.482156	F5/8 type C domain-containing protein
A0A2I5KI95	0.47887	Glycerol-3-phosphate dehydrogenase [NAD(P)+]
A0A4T2GKA7	0.477373	ATP-dependent zinc metalloprotease FtsH
A0A3R8LU71	0.472949	Uncharacterized protein
A0A0H3MW10	0.472508	Putative Mac family protein
D5AF31	0.471822	Probable endopeptidase lytE
A0A0Z8HVV7	0.465897	Uncharacterized protein
Q6XYR6	0.464025	Uncharacterized protein
A0A0Z8JEH8	0.456531	LPXTG-motif cell wall anchor domain-containing protein
A0A0Z8PZ88	0.44046	Chromosome segregation ATPase
A0A0Z8FJY1	0.438399	Glutamine ABC transporter substrate-binding protein
A0A0Z8N3W4	0.43787	LPXTG-motif cell wall anchor domain-containing protein
A0A075SKI3	0.437693	Nitroreductase
D5AIK0	0.434963	Amino acid ABC transporter, ATP-binding protein
D5AEY4	0.430925	GCN5-related N-acetyltransferase
A0A0Z8EWX1	0.42541	KxxxW cyclic peptide radical SAM maturase
A0A4T2H8B5	0.418882	YSIRK-type signal peptide-containing protein

A0A0Z8FP53	0.41877	Abortive infection bacteriophage resistance protein
A0A3R8NL30	0.410309	MFS transporter
A0A075SJX8	0.408687	PTS N-acetylgalactosamine transporter subunit IIA
A0A116LKE2	0.407618	Methyl-accepting chemotaxis protein
A0A4T2GGY9	0.392457	IS1182 family transposase
A0A116L790	0.391227	Type I restriction-modification system M protein
D5AHV5	0.385763	Response regulator consisting of a CheY-like receiver domain and a winged-helix DNA-binding domain
A0A4V0EKB7	0.380553	ATPase
A0A0M9FNZ7	0.379269	DEAD-box ATP-dependent RNA helicase CshB
G7SGA6	0.369637	GCN5-related N-acetyltransferase
A0A0Z8LG29	0.360294	Beta-glucosidase/6-phospho-beta-glucosidase/beta-galactosidase
A0A0Z8GVS0	0.357165	Transcriptional regulator
A0A0Z8JX73	0.337501	Phage protein
G7SDM4	0.334909	Uncharacterized protein
A0A426TDJ3	0.323642	DUF853 family protein
A4VXH0	0.321442	Clostridium cellulosome enzyme, dockerin type I
G7SLU7	0.305463	-
G7SNM8	0.276416	-
A0A0Z8E5D6	0.264391	Type I restriction modification DNA specificity protein
A0A116MN33	0.252316	OmpR family two component system phosphate regulon response regulator

TABLE S2 Sequence identity of the *ciaR*, *ciaH*, *satR*, *satA* and *satB* genes in *S. suis*.

S. suis strains	Gene sequence identity (%) [a]				
	ciaR	*ciaH*	*satR*	*satA*	*satB*
SC84	100	100	100	100	100
LSM102	100	100	100	100	100
ZY05719	100	100	100	100	100
98HAH33	100	99.71	100	100	100
A7	100	100	100	100	100
JS14	100	100	100	100	99.89
P1/7	100	100	100	100	100
SC070731	100	100	100	100	100
SS12	100	100	100	100	99.94
CS100322	100	100	100	100	100
BM407	99.7	97.32	100	100	100
ST1	99.7	97.32	84.44	96.31	95.76
S735	99.7	97.32	100	99.94	99.94
T15	98.81	96.52	99.33	96.19	96.36
NSUI002	98.21	96.3	82.93	86.55	95.13
NSUI060	98.21	96.38	82.93	86.55	95.07
HA0609	98.21	96.38	82.93	86.55	95.13
ST3	98.21	96.38	82.93	86.55	95.13
90-1330	98.21	96.38	82.93	86.55	95.13
YB51	98.21	96.38	82.93	86.55	95.13
05HAS68	98.21	96.38	82.93	86.55	95.07
D9	98.07	96.38	82.93	86.55	95.13
SRD478	97.62	96.09	84.04	85.33	90.94
ISU2812	94.65	97.32	93.99	96.19	95.24
SH1510	94.65	97.32	84.89	96.37	95.24
DN13	94.5	92.61	86.44	96.95	94.53
GZ0565	94.5	92.61	86.44	96.95	94.53
TL13	94.21	92.54	84.26	95.84	95.76
GZ1	94.21	92.61	100	100	100
D12	94.21	92.47	84.63	96.31	95.26
HN105	94.06	93.12	82.89	85.69	90.69
HA1003	94.05	92.47	82.48	85.66	91.08
1081	93.91	93.99	82.26	85.66	90.52
0061	93.76	92.25	82.26	85.66	90.52
CZ130302	91.98	91.96	87.08	94.49	94.12
AH681	87.82	92.4	91.45	95.49	93.4
HN136	87.67	92.4	93.32	95.31	93.46

a Gene sequence identity is obtained with other reported *S. suis* strains versus WT strains the *ciaR*, *ciaH*, *satR*, *satA* and *satB* genes of wild type strain.

TABLE S3 Primers used in this study.

Primers	Primers Sequence（5’-3’）	Description
For deletion		
satAB-L1	GCGTCGAC TTGTGTTTGAGAGCTTGTGCGACTT	Amplification of *satAB* upstream homology arm
satAB-L2	AACCTAGTGGCTGGGGTTTTCAACTGGCGTACCATTTATAACGTAAAATG	
satAB-R1	CATTTTACGTTATAAATGGTACGCCAGTTGAAAACCCCAGCCACTAGGTT	Amplification of *satAB* downstream homology arm
satAB-R2	CGGAATTCTTTCTTAGCGATGGTCATTTCCTCC	
satAB-IN1	ACAGAAAGGGAGGGAGGAATAGATG	Detection interior of *satAB* mutant strain
satAB-IN2	TCAAGGCAACTGGCGTAGAAGCAAT	
satAB-W1	CACCATTCACTTTTTTGGGGCATTC	Detection exterior of *satAB* mutant strain
satAB-W2	GTCGTAGAGATAGAGGACTTCGTTT	
satR-L1	GCGTCGACACCGTTGTCGTTGAGAACTTGTCCT	Amplification of *satR* upstream homology arm
satR-L2	TAAACATGATTCTTCTCCTTATAGCAGCCAAGTGCTCCACATCGTATTCT	
satR-R1	AGAATACGATGTGGAGCACTTGGCTGCTATAAGGAGAAGAATCATGTTTA	Amplification of *satR* downstream homology arm
satR-R2	CGGAATTCACCCGCATCAACACTGTAAAGAGCA	
satR-W1	ACAGTATGACCGTTGTCGTTGAGAA	Detection exterior of *satR* mutant strain
satR-W2	ATAGCCGATAAAGAGGTTGAGGTCC	
ciaR-L1	GCGTCGACTGCTATTTCAGTTGCTATCTTGGGC	Amplification of *ciaR* upstream homology arm
ciaR-L2	TAATTTTCCGATTCGCTTAGGCATTCATACACTTCCTTACGTTTCGGTTT	
ciaR-R1	AAACCGAAACGTAAGGAAGTGTATGAATGCCTAAGCGAATCGGAAAATTA	Amplification of *ciaR* downstream homology arm
ciaR-R2	CGGGATCCATTTTGAAGAACTGCCAACGGTGTG	
ciaR-IN1	GCGGATGTTATGCAGGTTTTTGATG	Detection interior of *ciaR* mutant strain
ciaR-IN2	CGACACTACGCAGGGTTTGAAGATT	
ciaH-L1	GCGTCGACCTGTCCCAGGTCTTTGGTTTGTTTA	Amplification of *ciaH* upstream homology arm
ciaH-L2	ACCTCTAATCTTGATGAATGGTTAGTTAGGCATTTTTTAGAATGTACCCG	
ciaH-R1	CGGGTACATTCTAAAAAATGCCTAACTAACCATTCATCAAGATTAGAGGT	Amplification of *ciaH* downstream homology arm

Primers	Primers Sequence（5’-3’）	Description
ciaH-R2	CGGGATCCCGACAGAAAAATAGACGCGCGAATA	
ciaH-IN1	AACCTATGAGCCCAACTCTGTCTTT	Detection interior of *ciaH* mutant strain
ciaH-IN2	AGATAAACCTAAACCAAATCCACCT	
ciaRH-W1	TAACATAATCAGGAGACTTCTGACG	Detection exterior of *ciaRH* mutant strain
ciaRH-W2	CAGTCAAACTCGAGTACTTCTTCTG	
ciaRH-IN1	TTCGAACTTCTTCTAAACTAGATCC	Detection interior of *ciaRH* mutant strain
ciaRH-IN2	GTCTATCAAATTCTGTTTTCGATTT	
For protein expression		
pCiaR-F	GGAATTCCATATGATGATTAAAATATTGTTAGTGG	Amplification of *ciaR* used in protein expression
pCiaR-R	CCGCTCGAGTTAATGGTGATGGTGATGGTGGGCATTTTTTAGAATGTACCCG	
pSatR-F	GGAATTCCATATGGGACATACTATTGCAGATTTTC	Amplification of *satR* used in protein expression
pSatR-R	CCGCTCGAGTTAATGGTGATGGTGATGGTG AACATGATTCTTCTCCTTATAG	
For complementation		
C*satR*-F	AAAACTGCAGAAAAAATCCTTTCTAGTTGGTGTAA	Amplification of *satR* and its promoter fragments
C*satR*-R	CGGGATCCTTAAACATGATTCTTCTCCTTATAG	
For checking satRAB operon		
SatRAB-TF1	CACCATTCACTTTTTTGGGGCATTC	Partial sequence of *satR* and *satA*
SatRAB-TR1	ACCATAGCCGATAAAGAGGTTGAGG	
SatRAB-TF2	AGCCAAGTAGAAGAACGTGGAAATA	Partial sequence of *satA* and *satB*
SatRAB-TR2	CAAGGAAAACAGGTGTCTTAACCTG	

Primers	Primers Sequence（5’-3’）	Description
For TSS		
CapRace-outer primer	AATGATACGGCGACCACCGAGATCTACACTCTTTCCCTACACGACGCTCTTCCGATCT	Amplification of first-round nest-PCR product
CapRace-satGSP1	ATTGCCCACCTGTTTAACCAAGTCA	
CapRace-inner primer	CTACACGACGCTCTTCCGATCT	Amplification of second-round nest-PCR product
CapRace-satGSP2	CTTTCAGGTGACATATCTTCCCTTG	
CapRace-TSO	ACACTCTTTCCCTACACGACGCTCTTCCGATCTrGrGrG	Template-switching oligonucleotide used to base pair with the extra cytosine residues of cDNA
For EMSA		
EMSA-*satRAB*-F	TTGACAATAGTTCTTAAGAGAACTATAATAGTTCTCATGGTAATTATTTTCGTAGAAAG	Formation of double stranded DNA probe by self-annealing
EMSA-*satRAB*-R	CTTTCTACGAAAATAATTACCATGAGAACTATTATAGTTCTCTTAAGAACTATTGTCAA	Target specific probe; the promoter of *satRAB*
EMSAneg-*satR*-F	CTCGCTTGGCCTTGGTGACCATTGCTTCTACGCCAGTTGCCTTGATTGCCTTGGTCGTT	Formation of double stranded DNA probe by self-annealing
EMSAneg-*satR*-R	AACGACCAAGGCAATCAAGGCAACTGGCGTAGAAGCAATGGTCACCAAGGCCAAGCGAG	The internal fragment of *satAB*
EMSAneg-*ciaR*-F	CGTGAAAAGGGGGTTACGACACCAGTTCTGATTACAACAGCCAAGGAAAGTCTAGAGGA	Formation of double stranded DNA probe by self-annealing
EMSAneg-*ciaR*-R	TCCTCTAGACTTTCCTTGGCTGTTGTAATCAGAACTGGTGTCGTAACCCCCTTTTCACG	The internal fragment of *ciaRH*
EMSApos-*ciaR*-F	AGGAATCAAGAAATTAAAGCTCAAA	Amplification of the promoter of *ciaR*
EMSApos-*ciaR*-R	CATACACTTCCTTACGTTTCGGTTT	
For qRT-PCR		
qPCR-*ciaR*-F	TAGTCATCAGCCCCCAATTC	Detection the transcription of *ciaR* used in qRT-PCR
qPCR-*ciaR*-R	GTTTATGAGGCGGAAACAGG	
qPCR-*ciaH*-F	CGCCTGGAAAGTCTATTTCG	Detection the transcription of *ciaH* used in qRT-PCR

Primers	Primers Sequence（5’-3’）	Description
qPCR-*ciaH*-R	TTCCATTTTCTTCGGCAATC	
qPCR-*satA*-F	CTACTTGGTGTCCGGGATGT	Detection the transcription of *satA* used in qRT-PCR
qPCR-*satA*-R	CATCCGCCACATCCTTGAAG	
qPCR-*satB*-F	TCTCGGGTACTATTGCGGAC	Detection the transcription of *satB* used in qRT-PCR
qPCR-*satB*-R	TTGTGGGTCTGTCAAAAGCG	
16S rRNA	GTTGCGAACGGGTGAGTAA	An internal region of 16S rRNA
	TCTCAGGTCGGCTATGTATCG	

Underlined are restriction cutting sites.

TABLE S4 Bacterial strains and plasmids used in this work.

Strain or Plasmid	Description[a]	Source or Reference
Strains		
SC19	From the brain of a dead pig	Our laboratory
Δ*ciaRH*	Deletion mutant of *ciaRH* with SC19 background	Our laboratory
Δ*ciaRH* Δ*satAB*	Deletion mutant of *satAB* with Δ*ciaRH* background	This study
Δ*ciaR*	Deletion mutant of *ciaR* with SC19 background	This study
Δ*ciaH*	Deletion mutant of *ciaH* with SC19 background	This study
Δ*satAB*	Deletion mutant of *satAB* with SC19 background	This study
Δ*satR*	Deletion mutant of *satR* with SC19 background	This study
Δ*ciaRH* Δ*satR*	Deletion mutant of *satR* with Δ*ciaRH* background	This study
Δ*ciaRH* C*satR*	Complemented strain of satR with Δ*ciaRH* background, Spc^r	This study
E. coli DH5α	Cloning host for maintaining the recombinant plasmids	TAKARA
E. coli BL21 (DE3)	Host for expressing the recombinant proteins	WEIDI
Plasmids		
pSET4S	Temperature-sensitive suicide vector, Spc^r	(1)
pSET2	vector for S. suis complementation, Spc^r	(2)
pET-30a (+)	vector for protein expression, Kan^r	

[a] Spc^r, spectinomycin resistant; Kan^r, kanamycin resistant.

REFERENCE

1. Takamatsu, D., Osaki, M., Sekizaki, T., 2001. Thermosensitive suicide vectors for gene replacement in *Streptococcus suis*. Plasmid 46, 140-148.

2. Takamatsu, D., Osaki, M., Sekizaki, T., 2001. Construction and characterization of *Streptococcus suis-Escherichia coli* shuttle cloning vectors. Plasmid 45, 101-113.
